# wQFM-TREE: highly accurate and scalable quartet-based species tree inference from gene trees

**DOI:** 10.1093/bioadv/vbaf053

**Published:** 2025-03-13

**Authors:** Abdur Rafi, Ahmed Mahir Sultan Rumi, Sheikh Azizul Hakim, Sohaib, Md Toki Tahmid, Rabib Jahin Ibn Momin, Tanjeem Azwad Zaman, Rezwana Reaz, Md Shamsuzzoha Bayzid

**Affiliations:** Department of Computer Science and Engineering, Bangladesh University of Engineering and Technology, Dhaka 1205, Bangladesh; Department of Computer Science and Engineering, Bangladesh University of Engineering and Technology, Dhaka 1205, Bangladesh; Department of Computer Science and Engineering, Bangladesh University of Engineering and Technology, Dhaka 1205, Bangladesh; Department of Computer Science and Engineering, Bangladesh University of Engineering and Technology, Dhaka 1205, Bangladesh; Department of Computer Science and Engineering, Bangladesh University of Engineering and Technology, Dhaka 1205, Bangladesh; Department of Computer Science and Engineering, Bangladesh University of Engineering and Technology, Dhaka 1205, Bangladesh; Department of Computer Science and Engineering, Bangladesh University of Engineering and Technology, Dhaka 1205, Bangladesh; Department of Computer Science and Engineering, Bangladesh University of Engineering and Technology, Dhaka 1205, Bangladesh; Department of Computer Science and Engineering, Bangladesh University of Engineering and Technology, Dhaka 1205, Bangladesh

## Abstract

**Motivation:**

Summary methods are becoming increasingly popular for species tree estimation from multi-locus data in the presence of gene tree discordance. Accurate Species TRee Algorithm (ASTRAL), a leading method in this class, solves the Maximum Quartet Support Species Tree problem within a constrained solution space, while heuristics like Weighted Quartet Fiduccia–Mattheyses (wQFM) and Weighted Quartet MaxCut (wQMC) use weighted quartets and a divide-and-conquer strategy. Recent studies showed wQFM to be more accurate than ASTRAL and wQMC, though its scalability is hindered by the computational demands of explicitly generating and weighting $\Theta (n^{4})$ quartets. Here, we introduce wQFM-TREE, a novel summary method that enhances wQFM by avoiding explicit quartet generation and weighting, enabling its application to large datasets.

**Results:**

Extensive simulations under diverse and challenging model conditions, with hundreds or thousands of taxa and genes, consistently demonstrate that wQFM-TREE matches or improves upon the accuracy of ASTRAL. It outperformed ASTRAL in 25 of 27 model conditions (statistically significant in 20) involving 200–1000 taxa. Moreover, applying wQFM-TREE to re-analyze the green plant dataset from the One Thousand Plant Transcriptomes Initiative produced a tree highly congruent with established evolutionary relationships of plants. wQFM-TREE’s remarkable accuracy and scalability make it a strong competitor to leading methods. Its algorithmic and combinatorial innovations also enhance quartet-based computations, advancing phylogenetic estimation.

**Availability and implementation:**

wQFM-TREE is freely available in open source form at https://github.com/abdur-rafi/wQFM-TREE.

## 1 Introduction

Inferring species trees from genes sampled throughout the whole genome is a fundamental problem in molecular evolutionary biology. However, this task is complicated by the phenomenon of gene tree discordance (or heterogeneity), suggesting that different parts of the genome may have different evolutionary histories due to various biological processes, including incomplete lineage sorting (ILS), gene duplication and loss, and horizontal gene transfer (Maddison 1997, Warnow 2017).

In the presence of gene tree heterogeneity, standard methods for estimating species trees, such as concatenation (which concatenates multiple sequence alignments of different genes into a single super-alignment and then estimates a tree from this alignment) can be statistically inconsistent when gene tree discordance is high due to ILS (Degnan *et al.* 2009, Roch and Steel 2015) and produce incorrect trees with high support (Kubatko and Degnan 2007). Therefore, “summary methods”, which operate by computing gene trees from different loci and then combining the inferred gene trees into a species tree, are becoming increasingly popular, and many of them are provably statistically consistent (Kubatko *et al.* 2009, Liu *et al.* 2009, Heled and Drummond 2010, Larget *et al.* 2010, Liu *et al.* 2010, Snir and Rao 2010, Liu and Yu 2011, Mossel and Roch 2011, Chifman and Kubatko 2014, Mirarab *et al.* 2014b, Reaz *et al.* 2014, Avni *et al.* 2015, Vachaspati and Warnow 2015, Islam *et al.* 2020, Mahbub *et al.* 2021).

Quartet-based summary methods have gained substantial attention as quartets (four-leaf unrooted gene trees) do not contain the “anomaly zone” (Degnan and Rosenberg 2006, 2009, Degnan 2013), a condition where the most probable gene tree topology may not be identical to the species tree topology. ASTRAL (Accurate Species TRee ALgorithm), the most popular summary method, is a dynamic programming algorithm that tries to solve the Maximum Quartet Support Species Tree (MQSST) problem (Mirarab *et al.* 2014b). It takes a set of gene trees as input and seeks to find a species tree so that the number of induced quartets in the gene trees that are consistent with the species tree is maximized. Weighted Quartet Fiduccia–Mattheyses (wQFM) (Mahbub *et al.* 2021) and Weighted Quartet MaxCut (wQMC) (Avni *et al.* 2015) are alternative heuristics to solve the MQSST problem. Both methods operate by deducing individual weighted quartets from gene trees and combining them into a cohesive species tree using a divide-and-conquer approach. In each divide step, taxa are partitioned based on the quartets derived from the input gene trees. The key distinction between wQFM and wQMC lies in the partitioning heuristic employed: wQFM utilizes the Fiduccia–Mattheyses (FM) heuristic (Fiduccia and Mattheyses 1982), while wQMC relies on MaxCut. wQFM and wQMC are extensions of the original QFM (Quartet Fiduccia–Mattheyses) (Reaz *et al.* 2014) and QMC (Quartet MaxCut) (Snir and Rao 2010, 2012) algorithms in order to support weighted quartets for construct phylogenetic trees. Notably, previous studies (Reaz *et al.* 2014, Mahbub *et al.* 2021, 2022) showed that QFM and wQFM are more accurate than QMC and wQMC. QFM is widely used in important phylogenetic studies (Mason *et al.* 2016, Georges *et al.* 2018, Malinsky *et al.* 2018, Devitt *et al.* 2019, Zhou *et al.* 2020, Foley *et al.* 2023), often in conjunction with the SVDquartets method (Chifman and Kubatko 2014). Although the heuristics themselves are computationally efficient, QFM/wQFM and QMC/wQMC rely on a quartet distribution rather than directly using gene trees. However, generating the quartet distribution is computationally expensive, requiring $\Theta (n^{4})$ time, for gene trees involving n taxa. This preprocessing step for generating quartet distributions and subsequent analyses become a significant bottleneck as the number of taxa increases, limiting the scalability of the methods for large datasets. Consequently, although wQFM has been shown to be more accurate or as good as ASTRAL (Mahbub *et al.* 2021), even when gene trees are incomplete (Mahbub *et al.* 2022), it has not received widespread attention from systematists. Therefore, addressing this bottleneck is crucial for making these algorithms practical for phylogenomic analyses involving thousands of taxa. In this context, Han and Molloy (2023) have pioneered an innovative approach, TREE-QMC, a substantially faster version of wQMC that operates directly on input gene trees, thereby eliminating the need to explicitly compute the weighted quartet distribution displayed by the input gene trees. Mim *et al.* (2023) have recently presented a fast implementation of the original QFM algorithm (Reaz *et al.* 2014), which solves the unweighted version of the maximum quartet consistency problem. In this work, inspired by Han and Molloy (2023), we introduce a novel method, wQFM-TREE (wQFM applied directly to gene trees), which employs an innovative technique to perform the same heuristic search as wQFM while eliminating the computational bottleneck of decomposing input gene trees into their induced quartets. With a time complexity of $O(n^{3}k\;\text{log}\;n)$ under certain assumptions (where n is the number of taxa and k is the number of gene trees), wQFM-TREE significantly improves the running time of wQFM, making it scalable to datasets with thousands of taxa and genes. Furthermore, consistent with observations from previous studies (Mahbub *et al.* 2021, 2022, Han and Molloy 2023) on small to moderate-sized datasets, wQFM-TREE exhibited remarkable accuracy on large datasets, surpassing ASTRAL on the majority of datasets analyzed in this study.

## 2 Materials and methods

We begin with a brief overview of the original wQFM. We next describe our proposed techniques introduced in wQFM-TREE that enable its direct application to gene trees.

### 2.1 Background on wQFM

Given a set $G=\{g_{1},g_{2},\ldots ,g_{k}\}$ of k gene trees on taxa set X, wQFM computes weights for every possible quartet ab|cd, where ab|cd denotes the unrooted quartet tree with leaf set a,b,c,d∈X in which the pair a,b is separated from the pair c,d by an edge. These weighted quartets are used in a divide-and-conquer algorithm to construct a species tree. In this approach, the algorithm first produces a bipartition of X based on the weighted quartets using the FM heuristic (Fiduccia and Mattheyses 1982), recursively calculates rooted trees on each subset of the bipartition, and then combines the rooted trees to form a complete species tree on the full taxa set (Supplementary Fig. 1).

Central to wQFM’s search algorithm is generating k  $\left(\begin{array}{c} n\\ 4 \end{array}\right)$ quartets induced by the input gene trees, computing the weights of these induced quartets based on gene tree frequency, and scoring a candidate bipartition based on the weights of the input quartets that are consistent and conflicting with that particular bipartition. Generating weighted quartets (and saving them to a file using appropriate I/O operations) is an extremely time and memory-consuming step and constitutes a significant portion of wQFM’s overall runtime. This weighted quartet generation step has actually been the main limitation for the broader application of wQFM to larger datasets.

Each divide step of the wQFM algorithm splits the set of taxa into two disjoint subsets (a bipartition of the taxa set, each representing a subproblem). A unique “dummy taxon” is added to both of the subsets so that the solutions to the subproblems can be combined within a divide-and-conquer framework (Supplementary Fig. 1). The technique to find a bipartition begins with an initial bipartition, followed by a heuristic iterative strategy to improve it. Within this iterative process, each iteration begins with the bipartition from the previous iteration and tries to improve it by moving taxa from one partition to another. The iterative improvement strategy is described in detail in Reaz *et al.* (2014). The effectiveness of such a move, which we call “Gain”, is quantified by the differences in bipartition scores before and after a taxon transfer. Mim *et al.* (2023) showed that given $\Theta (n^{4})$ quartets, one iteration of this technique to improve an initial bipartition takes $O(n^{4})$ time, which is very high. Producing an initial bipartition at every bipartitioning step is also a highly time-consuming process. The computation of the initial bipartition entails working with sorted weighted quartets, aiming to identify a bipartition consistent with quartets of substantial weights. Sorting $\Theta (n^{4})$ weighted quartets and selecting a good initial bipartition accordingly is a highly time-consuming step.

### 2.2 Overview of wQFM-TREE

Han and Molloy (2023) developed an innovative approach to applying the QMC algorithm directly to gene trees. Building on their concept, we have developed new techniques to enable QFM to operate on gene trees without requiring decomposition into induced quartets. While our overall framework draws inspiration from Han and Molloy (2023), the underlying algorithmic and combinatorial methodologies differ significantly. This is because FM, which is used in QFM and wQFM, and MaxCut (used in QMC) are fundamentally distinct algorithms, requiring specialized theoretical foundations and algorithmic adaptations to extend QFM and wQFM for direct application to gene trees. For example, unlike QMC, QFM and wQFM begin each divide step with an initial bipartition, which is iteratively refined using the FM algorithm. In the original QFM/wQFM framework, constructing the initial bipartition involves processing sorted weighted quartets to identify a bipartition that aligns with quartets of significant weights. However, generating the $\Theta (n^{4})$ weighted quartets, sorting them, and subsequently determining a suitable initial bipartition is an extremely time-intensive process. In wQFM-TREE, we utilized a consensus tree-based approach to find an initial bipartition directly from gene trees. In QMC and TREE-QMC, score calculation relies on determining the number of “good” and “bad” edges in the “quartet graph” (Snir and Rao 2012, Han and Molloy 2023), with TREE-QMC introducing an innovative technique to compute these directly from gene trees. In contrast, QFM and wQFM do not utilize a quartet graph. Instead, the underlying FM algorithm requires computing the score of candidate bipartitions during the iterative improvement of the initial bipartition. To address this, our proposed wQFM-TREE method introduces novel algorithmic techniques to directly derive these scores from the gene trees, eliminating the time-consuming step of generating and sorting quartets. Consequently, the approaches and techniques in wQFM-TREE are substantially different from those employed in TREE-QMC.

In particular, wQFM-TREE employs the following two key strategies: (i) using a gene tree consensus-based method to compute an initial bipartition for each divide step (see Section 2.4 and Algorithm 1 in Supplementary Section 1) and (ii) utilizing combinatorial and graph theoretic techniques to compute the scores of candidate bipartitions (Section 2.5 and Algorithm 2 in Supplementary Section 1) directly from gene trees, thereby eliminating the need for generating and computing the weights of the induced quartets. Both of these strategies heavily depend on a certain tree structure that we define for dummy taxa (discussed in Section 2.3) in our proposed method. One iteration of the iterative improvement strategy for finding a bipartition takes $O(n^{2}k\;\text{log}\;n)$ with our proposed techniques instead of the $O(n^{4})$ runtime of the original wQFM, with certain assumptions on subproblem sizes (details in Supplementary Section 3).

### 2.3 Tree structure of a dummy taxon and weighting scheme

**Figure 1.**
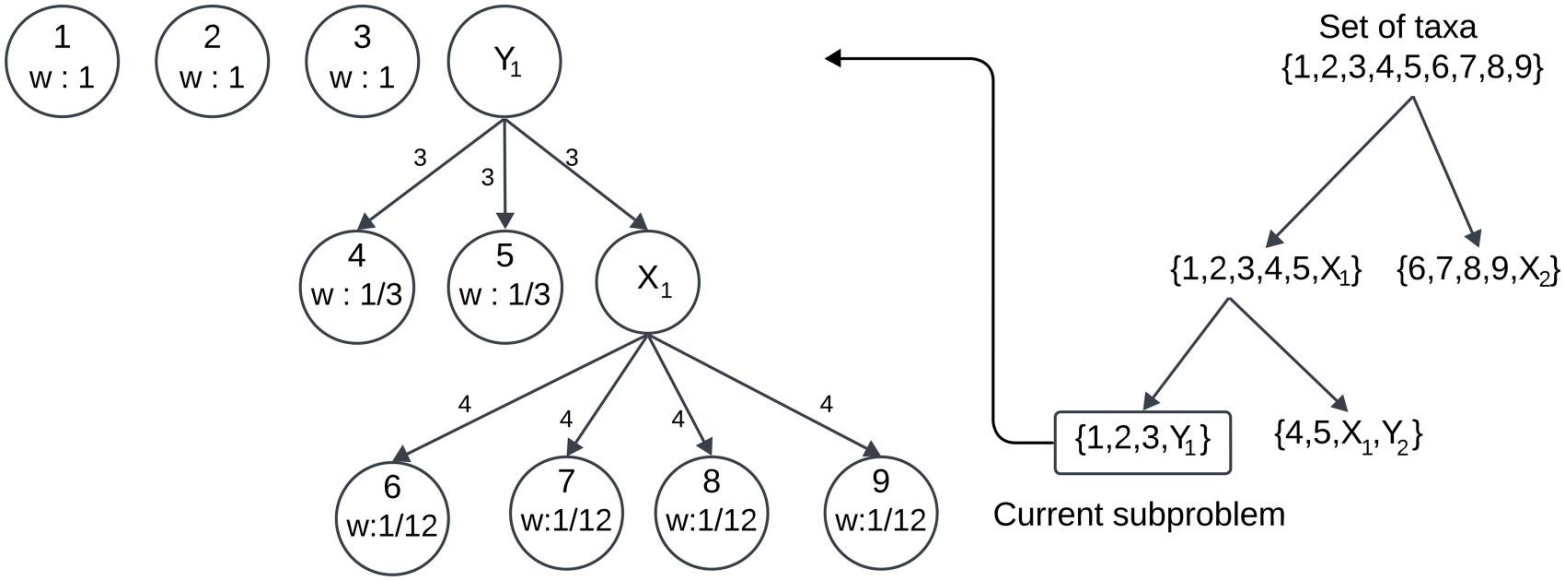
Tree structure of a dummy taxon, and the assignment of weights to real taxa under this dummy taxon. Here, $Y_{1}$ is a dummy taxon that represents $\left\{4,5,X_{1}\right\}$, where $X_{1}$ is another dummy taxon representing {6,7,8,9}. The entire tree structure (root labeled by $Y_{1}$) is the representation of dummy taxon $Y_{1}$. The real taxa under $Y_{1}$ appear as leaves in the tree structure. Edge weights for each edge are shown. The weight of a leaf is the multiplicative inverse of the product of the edge weights from the root to the leaf.

In our divide step, we create a bipartition (A,B) of the taxa set and create two new subproblems, one with taxa set $A\cup \{X_{1}\}$ and the other with taxa set $B\cup \{X_{2}\}$, where $X_{1}$ and $X_{2}$ are dummy taxa. Note that, unlike the original wQFM, we differentiate between the dummy taxa added to the two subproblems. $X_{1}$ essentially represents the taxa in B, and $X_{2}$ represents the taxa in A. We model $X_{1}$ and $X_{2}$ as rooted tree structures, where the children of the roots are the taxa in B and A, respectively. If *A, B* contain any dummy taxon, they will appear as subtrees in the tree structure of $X_{2},X_{1}$, respectively, resulting in a recursive tree structure (Fig. 1). Therefore, the tree representation of a dummy taxon X is a rooted tree with height >1. The leaves of this tree are real taxa, and any internal node is a dummy taxon, which was introduced in an earlier subproblem. We say that the leaves or real taxa in the tree structure of X are “under dummy taxon” X and denote this set of real taxa under X as $X_{R}$.

Now we describe how we assign weight w(a) to a taxon a that is used to compute the score of a candidate bipartition as described in Section 2.5. We assign unit weight to the real taxa that are not under any dummy taxa of the current subproblem. Note that the contribution of a dummy taxon to the score of a candidate bipartition should be equal to that of a real taxon as both can be considered equivalent in a subproblem. Therefore, for a dummy taxon X, we assign fractional weights to the real taxa in $X_{R}$ in such a way that $w(X)=\sum_{a\in X_{R}}w(a)=1$. We refer to this assignment as weight normalization, and we describe our motivation behind it in more detail in Supplementary Section 2.3. The normalization process is as follows. We first assign weights to the edges of the tree structure corresponding to X. We then use these edge weights to calculate w(a) ($a\in X_{R}$). Refer to Fig. 1 for an example. The weight of an edge (u,v) with u as the parent node is defined as the number of children of u. Let $p_{a}$ be the set of edges in the path from root to a. We define w(a) as ${\left(\prod_{(u,v)\in p_{a}}w_{\left(u,v\right)}\right)}^{-1}$. Therefore, for any real taxon a, w(a) is defined as follows:


(1) $$w(a)=\{\begin{array}{cc} {\left(\prod_{(u,v)\in p_{a}}w_{\left(u,v\right)}\right)}^{-1} & \text{if}\;a\;\text{is}\;\text{under}\;a\;\text{dummy}\;\text{taxon}\\ 1 & \text{otherwise} \end{array}$$

We note that this weighting mechanism was originally proposed and used in the TREE-QMC method (Han and Molloy 2023) for weighting quartets to normalize the “Quartet Graph”. We use this weighting mechanism with a slightly different motivation than the TREE-QMC method for the following use cases: (i) to construct initial bipartition from consensus tree of gene trees and (ii) to calculate the score of a candidate bipartition using weighted satisfied and violated quartets.

### 2.4 Computing an initial bipartition for a divide step

Let S be the set of taxa of a subproblem in a divide step of our method. We need to create an initial bipartition of S, which will subsequently be improved in an iterative manner. To do so, we create and utilize a greedy consensus tree of the input gene trees, also known as the majority rule extended tree, using PAUP v4.0b10 (Swofford 2002). This works even for incomplete gene trees, which are very common in real datasets.

Each bipartition (A,B) corresponding to each edge of the consensus tree defines a bipartition $\left(S_{A},S_{B}\right)$ of S as follows. S may contain both real and dummy taxa. The real taxa in S are partitioned according to (A,B) meaning that if a taxon s∈A, then $s\in S_{A}$. We now describe how a dummy taxon X in S is assigned to a partition ($S_{A}$ or $S_{B}$). We compute the weight of each taxon in $X_{R}$ (the set of real taxa present in the tree structure of X) as described in Section 2.3. $X_{R}$ can be partitioned to $S_{A}$ and $S_{B}$ according to (A,B), similar to how we partition the real taxa in S with respect to (A,B). Next, we assign X to the partition $S_{A}$ if the sum of weights of the real taxa in $X_{R}$ that belong to A is greater than the sum of weights of the real taxa in $X_{R}$ that belong to B. Otherwise, we assign X to the partition $S_{B}$. Thus, we find a bipartition $\left(S_{A},S_{B}\right)$ of S with respect to (A,B) in the consensus tree. Next, we score $\left(S_{A},S_{B}\right)$ using the scoring scheme described in Section 2.5. We find and score all such bipartitions of S corresponding to the bipartitons in the consensus tree. We choose the bipartition of S with the highest score as the initial bipartition. The pseudocode for this algorithm is presented as Algorithm 1 in the Supplementary Material.

### 2.5 Scoring a candidate bipartition directly from gene trees

Let G be a set of unrooted gene trees on taxa set X. We allow the gene trees to be non-binary and incomplete (i.e. some taxa could be missing in a gene tree). Let $X^{\left(g\right)}$ be the set of real taxa in a gene tree g∈G. Let (A,B) be the given bipartition. Let $R_{A}$ and $D_{A}$ be the sets of real and dummy taxa in A, respectively. Let $F_{A}$ be the set of all real taxa that are either in $R_{A}$ or under a dummy taxon $X\in D_{A}$, i.e. $F_{A}=(\underset{X\in D_{A}}{\cup }X_{R})\cup R_{A}$. We define $R_{B},D_{B},F_{B}$ similarly.

Our algorithm assigns weight to each real taxon in X according to (1). These weights are used to calculate the weights of quartets. Let a,b,c,d∈X. We define the weight of an unordered pair of taxa, p={a,b} as w(p)=w(a)·w(b). We consider a quartet q=ab|cd to be composed of two unordered pairs {a,b} and {c,d}. We define its weight w(ab|cd) to be w({a,b})·w({c,d}). We define the weight of a set Q of quartets as $w(Q)=\sum_{q\in Q}w(q).$ We define the weight of a set of unordered pairs similarly.

Given a bipartition (A,B), each quartet ab|cd where no two taxa in {a,b,c,d} are under the same dummy taxon of the current subproblem, can be classified as one of the following three types.

1. **Satisfied:** where either $\{a,b\}\subseteq F_{A}$ and $\{c,d\}\subseteq F_{B}$ or $\{a,b\}\subseteq F_{B}$ and $\{c,d\}\subseteq F_{A}$
2. **Deferred:** where $P\subseteq F_{A}$ or $P\subseteq F_{B}$, P⊆{a,b,c,d}, and |P|≥3.
3. **Violated:** all other quartets.

Note that we do not consider the quartets that have at least two taxa under the same dummy taxon. This is because although a dummy taxon may have multiple real taxa under it, it is considered a single taxon for the current subproblem. Therefore, a dummy taxon should not contribute more than once in a quartet. In the original wQFM, we do not need to consider this extra condition as it enumerates all the quartets and updates the set of considered quartets for each subproblem in the divide step.

We note that if a gene tree g is non-binary, then for some $a,b,c,d\in X^{\left(g\right)}$, a quartet structure may not be formed/resolved (i.e. there is no branch separating two taxa from the other two). We call such quartets “unresolved”.

Let $S^{\left(g\right)},V^{\left(g\right)}$ be the sets of satisfied and violated resolved quartets respectively in a gene tree g∈G. Then the score Score(A,B,G) of a candidate bipartition (A,B) with respect to G is defined as $\mathrm{Score}(A,B,G)=\sum_{g\in G}(w(S^{\left(g\right)})-w(V^{\left(g\right)}))$.

Let $U^{\left(g\right)}$ be the set of satisfied or violated unresolved quartets. Since $S^{\left(g\right)}$, $V^{\left(g\right)}$, $U^{\left(g\right)}$ are disjoint sets, $w(S^{\left(g\right)}\cup V^{\left(g\right)}\cup U^{\left(g\right)})=w(S^{\left(g\right)})+w(V^{\left(g\right)})+w(U^{\left(g\right)})$. We can rewrite $\left(w(S^{\left(g\right)})-w(V^{\left(g\right)})\right)$ as $\left(2w(S^{\left(g\right)})-(w(S^{\left(g\right)})+w(V^{\left(g\right)})+w(U^{\left(g\right)}))+w(U^{\left(g\right)})\right)$. Thus, we get the following equation:


$$\begin{array}{c} \mathrm{Score}(A,B,G)\\ =\sum_{g\in G}(2w(S^{\left(g\right)})-w(S^{\left(g\right)}\cup V^{\left(g\right)}\cup U^{\left(g\right)})+w(U^{\left(g\right)})) \end{array}$$

We do such restructuring of this equation for computational efficiency. Calculating $w(S^{\left(g\right)})$ and $w(V^{\left(g\right)})$ separately requires traversing each internal node of the tree g and perform certain calculations (details in Section 2.5.1 for $w(S^{\left(g\right)})$). However, $w(S^{\left(g\right)}\cup V^{\left(g\right)}\cup U^{\left(g\right)})$ can be calculated directly from $X^{\left(g\right)}$ (details in Section 2.5.2). Hence, by eliminating $w(V^{\left(g\right)})$ and incorporating $w(S^{\left(g\right)}\cup V^{\left(g\right)}\cup U^{\left(g\right)})$ into the equation, the computation becomes notably faster in practice. Although we have to compute $w(U^{\left(g\right)})$ in this approach, it is still more efficient because calculating $w(U^{\left(g\right)})$ requires us to consider only the polytomy nodes (details in Section 2.5.3). The pseudocode for scoring a candidate bipartition is presented as Algorithm 2 in the Supplementary Material.

#### 2.5.1 Computing $w(S^{\left(g\right)})$, the sum of weights of satisfied resolved quartets in a gene tree g

Let u be an internal node in g and deg(u) denote the degree of u. Removing u will create deg(u) components of g. We define $C^{\left(g,u\right)}$ to be the set of these components and use $C_{i}^{\left(g,u\right)},1\leq i\leq \deg(u)$ to refer to the $i^{th}$ component. We say, a real taxon $a\in C_{i}^{\left(g,u\right)}$ if a is in component $C_{i}^{\left(g,u\right)}$.

The subtree of a fully resolved tree restricted to a quartet exhibits two degree-three nodes. We refer to these nodes as “anchors” of the quartet on that tree. Now, let $S^{\left(g,u\right)}$ be the set of all resolved satisfied quartets ab|cd such that $a,b\in F_{A}$, $c,d\in F_{B}$, and a,b belong to two distinct components in $C^{\left(g,u\right)}$ and both c,d pertain to a third component of $C^{\left(g,u\right)}$. We observe that $S^{\left(g,u\right)}$ actually contains the quartets, which have u as the anchor directly connected to a,b in the quartet tree. All such sets $S^{\left(g,u\right)}$, across every internal node u, form a partition of $S^{\left(g\right)}$ (Lemma 1 in Supplementary Section 2). As a result, $w(S^{\left(g\right)})=\sum_{u}w(S^{\left(g,u\right)})$.

To compute $w(S^{\left(g,u\right)})$, we further split $S^{\left(g,u\right)}$ into disjoint sets $S_{i,j,k}^{\left(g,u\right)},1\leq i,j,k\leq \deg(u),i<j,k\neq i,j.$  $S_{i,j,k}^{\left(g,u\right)}$ contains the satisfied quartets ab|cd where $a,b\in F_{A},a\in C_{i}^{\left(g,u\right)},b\in C_{j}^{\left(g,u\right)}$ and $c,d\in F_{B},c,d\in C_{k}^{\left(g,u\right)}.$ Let $PA_{i,j}^{\left(g,u\right)}$ be the set of unordered pairs {a,b} where a,b are not under the same dummy taxon, $a,b\in F_{A}$, and $a\in C_{i}^{\left(g,u\right)},b\in C_{j}^{\left(g,u\right)}$. Similarly, let $PB_{k}^{\left(g,u\right)}$ be the set of unordered pairs {c,d} where c,d are not under the same dummy taxon, $c,d\in F_{B}$, and $c,d\in C_{k}^{\left(g,u\right)}$, c≠d. Therefore, for a quartet $ab|cd\in S_{i,j,k}^{\left(g,u\right)}$, the unordered pair $\{a,b\}\in PA_{i,j}^{\left(g,u\right)}$ and the unordered pair $\{c,d\}\in PB_{k}^{\left(g,u\right)}$ where k≠i,j. As a result, $w(S_{i,j,k}^{\left(g,u\right)})=w(PA_{i,j}^{\left(g,u\right)})w(PB_{k}^{\left(g,u\right)})$. Then we compute 


$$w(S^{\left(g,u\right)})=\sum_{i<j}\sum_{k\neq i,j}S_{i,j,k}^{\left(g,u\right)}$$

We restructure this expression for efficient implementation, which is described in Supplementary Section 2.1.3. We can calculate $w(PA_{i,j}^{\left(g,u\right)})$, $w(PB_{k}^{\left(g,u\right)})$ without enumerating the sets $PA_{i,j}^{\left(g,u\right)}$, $PB_{k}^{\left(g,u\right)}$ (see Supplementary Section 2.1 for details). Thus, for a given candidate bipartition (A,B), we can compute the weight of the satisfied resolved quartets in g without explicitly enumerating its induced set of quartets.

#### 2.5.2 Computing $w(S^{\left(g\right)}\cup V^{\left(g\right)}\cup U^{\left(g\right)})$, the sum of weights of satisfied or violated quartets in g

We notice that, each quartet $q=ab|cd\in (S^{\left(g\right)}\cup V^{\left(g\right)}\cup U^{\left(g\right)})$ has two taxa from $F_{A}$, the other two from $F_{B}$ and no pair of taxon from {a, b, c, d} is under the same dummy taxon. Let $PA^{\left(g\right)}$ be the set of unordered pairs {a,b} where a,b are not under the same dummy taxon and $a,b\in F_{A}\cap X^{\left(g\right)}$ (the intersection with $X^{\left(g\right)}$ allows our computation to be applicable for incomplete gene trees where some taxa are missing). Similarly, let $PB^{\left(g\right)}$ be the set of pairs {c,d} where c,d are not under the same dummy taxon and $c,d\in F_{B}\cap X^{\left(g\right)}$. Each pair $\{a,b\}\in PA^{\left(g\right)}$ forms one quartet with each pair $\{c,d\}\in PB^{\left(g\right)}$. Thus, we obtain $w(S^{\left(g\right)}\cup V^{\left(g\right)}\cup U^{\left(g\right)})=w(PA^{\left(g\right)})\cdot w(PB^{\left(g\right)})$. We provide the expressions for calculating $w(PA^{\left(g\right)})$ and $w(PB^{\left(g\right)})$ in Supplementary Section 2.1.4.

#### 2.5.3 Computing $w(U^{\left(g\right)})$, the sum of weights of unresolved satisfied or violated quartets in g

For each $ab|cd\in U^{\left(g\right)}$, we can find only one internal node u in g such that a,b,c,d, all come from four different components in $C^{\left(g,u\right)}$ and $a,b\in F_{A}$, $c,d\in F_{B}$. Let $U^{\left(g,u\right)}$ be the set of these quartets. We obtain $w(U^{\left(g\right)})$ by adding $w(U^{\left(g,u\right)})$ for each polytomy node u.

Similar to computing $w(S^{\left(g,u\right)})$, we further split $U^{\left(g,u\right)}$ into disjoint sets $U_{i,j,k,l}^{\left(g,u\right)},1\leq i,j,k,l\leq \deg(u),i<j,k<l,k\neq i,j;l\neq i,j.$  $U_{i,j,k,l}^{\left(g,u\right)}$ contains the unresolved quartets ab|cd of $U^{\left(g,u\right)}$ where $a\in C_{i}^{\left(g,u\right)}$,$b\in C_{j}^{\left(g,u\right)}$,$c\in C_{k}^{\left(g,u\right)}$, and $d\in C_{l}^{\left(g,u\right)}$. Let $PB_{k,l}^{\left(g,u\right)}$ be the set of unordered pairs {c,d}, where c,d are not under the same dummy taxon, $c,d\in F_{B}$, and $c\in C_{k}^{\left(g,u\right)},d\in C_{l}^{\left(g,u\right)}$. Note that each pair $\{a,b\}\in PA_{i,j}^{\left(g,u\right)}$ forms an unresolved quartet with each pair $\{c,d\}\in PB_{k,l}^{\left(g,u\right)}$ when k<l,k≠i,j;l≠i,j and these quartets form the set $U_{i,j,k,l}^{\left(g,u\right)}$. Therefore $w(U_{i,j,k,l}^{\left(g,u\right)})=w(PA_{i,j}^{\left(g,u\right)})w(PB_{k,l}^{\left(g,u\right)})$. Then


$$\begin{array}{c} w(U^{\left(g,u\right)})=\sum_{i<j}\sum_{k<l;k,l\notin \{i,j\}}w(U_{i,j,k,l}^{\left(g,u\right)})\\ =\sum_{i<j}w(PA_{i,j}^{\left(g,u\right)})(\sum_{k<l;k,l\notin \{i,j\}}w(PB_{k,l}^{\left(g,u\right)})) \end{array}$$

We employ this equation to derive an expression for $w(U^{\left(g,u\right)})$ that enables efficient implementation (Supplementary Section 2.1.5). We present an example showing detailed calculations of our scoring method in Supplementary Section 2.2.

#### 2.5.4 Gain calculation

Note that the wQFM algorithm improves a bipartition iteratively by transferring taxa from one partition to the opposite, and the effectiveness of a move is quantified by “Gain”, which is the change of scores after transferring a taxon. The procedure for calculating gains using our scoring method is outlined in Supplementary Section 2.4.

### 2.6 Time complexity

Assuming that the input gene trees are fully resolved or polytomies are resolved arbitrarily, and the subproblems are produced in a perfectly balanced fashion, wQFM-TREE can run in $O(n^{3}k\;\text{log}\;n)$ time, where n and k denote the number of taxa and gene trees, respectively. A detailed time complexity analysis is presented in Section 3 of the Supplementary Materials. We note that TREE-QMC has a time complexity of $O(n^{3}k)$ considering balanced subproblems (TREE-QMC needs to resolve polytomies arbitrarily).

## 3 Experimental studies

### 3.1 Datasets

#### 3.1.1 Simulated dataset

We evaluated wQFM-TREE on large-scale simulated datasets from the ASTRAL-II study (Mirarab and Warnow 2015). We used eight model conditions from the ASTRAL-II dataset, simulated under the Yule model from model species trees, which are characterized by three parameters: the height of the species tree, the speciation rate, and the number of taxa. In six model conditions, the number of taxa was set to 200 with varying tree lengths (500 K, 2 M, and 10 M generations) and speciation rates (1e-6 and 1e-7 per generation). The remaining two model conditions contain 500 and 1000 taxa with tree shapes fixed to 2 M/1e-6. For all the model conditions, we assessed the performance on varying the number of genes (50, 200, and 1000). Each model condition has 50 replicates of data. We analyzed all 50 replicates except for the 1000 taxa dataset, for which we analyzed 20 replicates (R1−R21, excluding *R*8) in our study. We excluded *R*8 as wQFM-TREE failed to analyze it in the model condition with 1000 gene trees within the allotted 48 h of computation due to the presence of high levels of polytomies. However, upon resolving the polytomies, wQFM-TREE successfully analyzed this replicate in just 4 h. A discussion on handling polytomies is provided in Section 4.1.2.

Moreover, we compared wQFM-TREE to the wQFM on the 48-taxon avian and 37-taxon mammalian simulated datasets from (Mirarab *et al.* 2014a) as the original wQFM cannot handle large datasets with hundreds of taxa. These datasets were simulated based on previously published species trees for 48 birds (Jarvis *et al.* 2014) and 37 mammals (Song *et al.* 2012), respectively. We also analyzed a 2000-taxon dataset from the ASTRAL-III study (Zhang *et al.* 2018) to test the scalability of wQFM-TREE.

#### 3.1.2 Empirical dataset

We re-analyzed the green plant dataset (1 kp, 2019)—one of the largest phylogenomic datasets analyzed so far—from the One Thousand Plant Transcriptomes Initiative (2019). This dataset contains 410 single-copy gene family trees across 1178 species, including green plants (Viridiplantae), glaucophytes (Glaucophyta), red algae (Rhodophyta), and outgroup species. Moreover, to demonstrate the scalability of wQFM-TREE on very recent large datasets, we applied wQFM-TREE to the extended avian dataset (Stiller *et al.* 2024) containing 363 species and 14 972 exon loci.

### 3.2 Methods and measurements

We compared wQFM-TREE to the best existing species tree estimation methods wQFM, ASTRAL-III (v.5.7.8), and TREE-QMC.

On the simulated datasets, we compared the estimated trees with the model species tree using normalized Robinson–Foulds (RF) distance (Robinson and Foulds 1981). For the biological dataset, we compared the estimated species trees to established evolutionary relationships. We assessed support values in the estimated trees using local posterior probabilities (Sayyari and Mirarab 2016) computed by ASTRAL. We analyzed multiple data replicates under various model conditions and conducted a two-sided Wilcoxon signed-rank test (Wilcoxon 1945) to compare the distribution of RF rates across different replicates in a particular model condition. We set the threshold α=0.05 to measure the statistical significance of the differences between two methods.

## 4 Results and discussion

### 4.1 Results on simulated datasets

We first compare wQFM-TREE with wQFM in terms of accuracy and running time. Next, we extensively compare wQFM-TREE with ASTRAL and TREE-QMC on large datasets with hundreds of taxa and genes. Due to space constraints, the experimental results comparing wQFM-TREE and wQFM are presented in Supplementary Section 4.1. These results indicate that wQFM-TREE is significantly faster than the original wQFM without sacrificing the accuracy.

#### 4.1.1 Comparing wQFM-TREE with other coalescent-based methods

##### 4.1.1.1 Results on 200-taxon dataset

**Figure 2.**
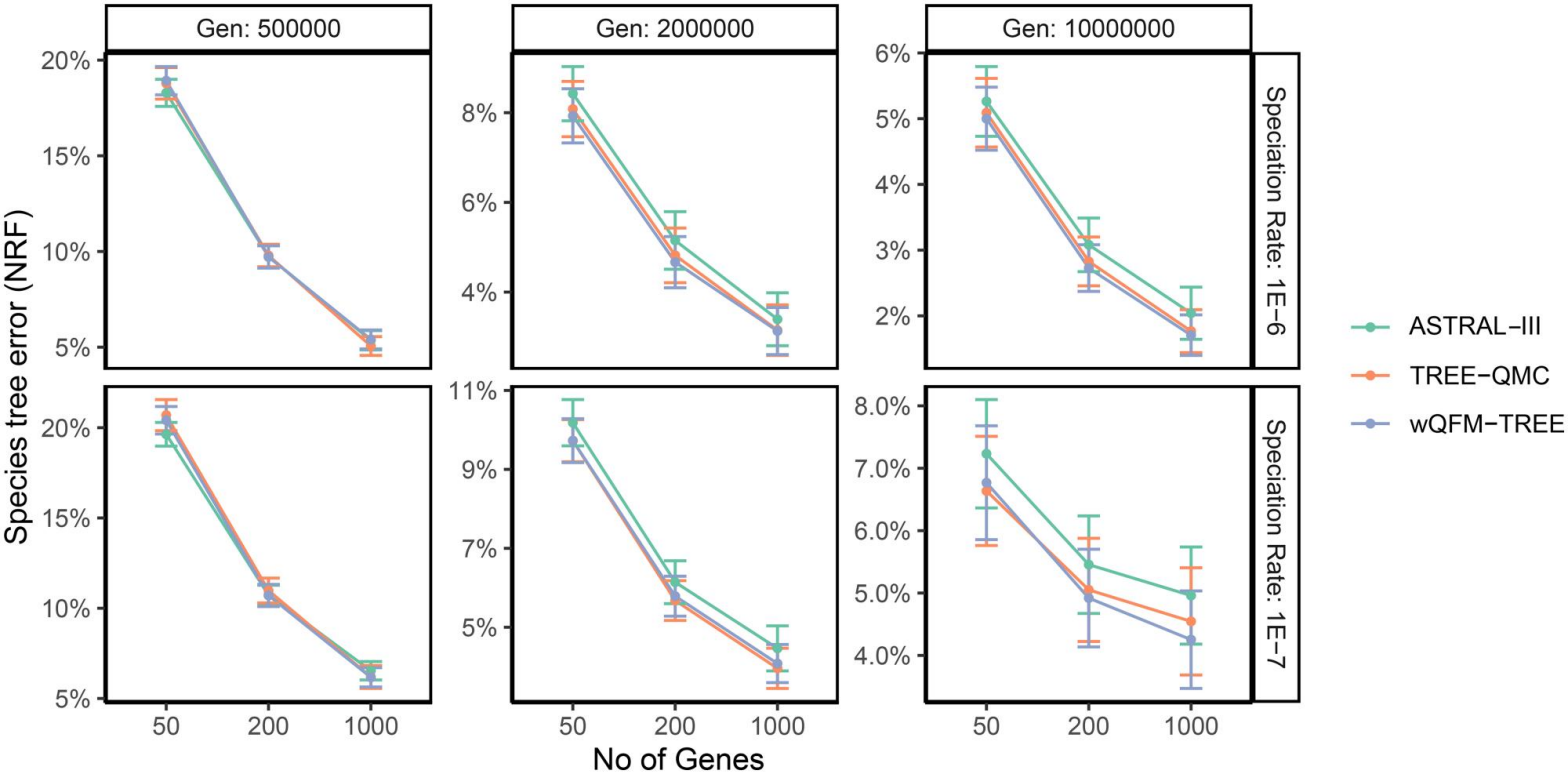
Comparison of methods on 200-taxon dataset with varying tree shapes and numbers of genes.

The evaluation of wQFM-TREE, ASTRAL, and TREE-QMC is presented in Fig. 2 for the 200-taxon datasets under various model conditions with varying tree lengths, speciation rates, and numbers of taxa. On relatively short tree lengths (i.e. 500K generations), wQFM-TREE performed comparably with ASTRAL and the differences between the methods were often statistically insignificant. wQFM-TREE performed better than ASTRAL with statistical significance in one model condition and so did ASTRAL in two model conditions. However, as the number of generations increased, wQFM-TREE demonstrated superior performance. Specifically, wQFM-TREE outperformed ASTRAL in all 12 model conditions with 2M and 10M generations, and the improvements were statistically significant (P<0.05) in 11 of these 12 model conditions. Additionally, the improvements of wQFM-TREE over ASTRAL increased with increasing numbers of genes.wQFM-TREE and TREE-QMC performed very closely on this particular dataset, with most of the differences being not statistically significant. Among the 18 model conditions examined, wQFM-TREE was better than TREE-QMC on 11 model conditions, with statistically significant differences in 2 of these 11 model conditions. On the other hand, TREE-QMC was better than wQFM-TREE on six model conditions, with improvements being statistically significant in two instances. The *P*-values from the statistical significance tests are provided in Supplementary Section 4.3.

##### 4.1.1.2 Results on varying numbers (200, 500, and 1000) of taxa

**Figure 3.**
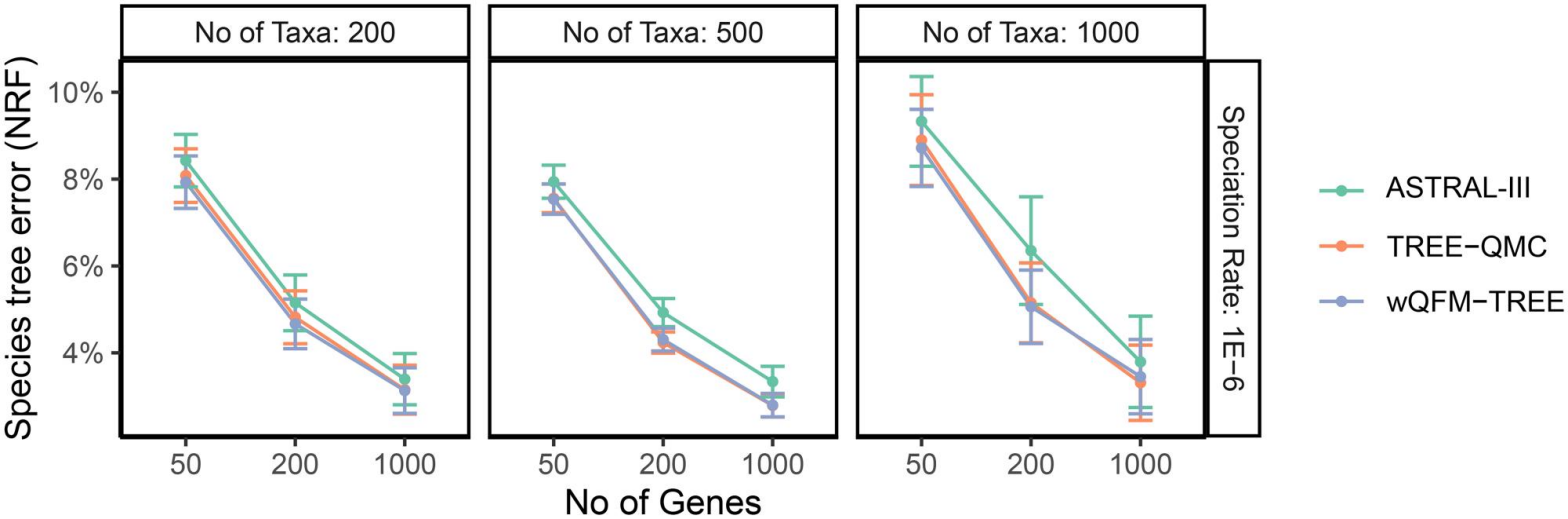
Comparison of methods on varying numbers of taxa (200, 500, and 1000) and genes (50, 200, and 1000) with the tree shape fixed to 2M/1e-6.

Figure 3 shows the assessment of different methods on varying the number of taxa (200, 500, and 1000) and genes (50, 200, and 1000) with the tree shape fixed to 2 M/1e-6. Remarkably, wQFM-TREE outperformed ASTRAL in all the model conditions, with differences being statistically significant (P<0.05) in nine of the nine model conditions. Notably, the superiority of wQFM-TREE over ASTRAL became more pronounced with the increasing number of taxa. It is important to note that ASTRAL is a highly accurate and the most widely used method for species tree estimation. Therefore, the demonstrated consistent improvement over ASTRAL, albeit small in some cases, is remarkable. wQFM-TREE and TREE-QMC showed comparable performance, with no statistically significant difference except in one model condition where wQFM-TREE was better with statistical significance. The *P*-values from the statistical significance tests are presented in Supplementary Section 4.3.

**Figure 4.**
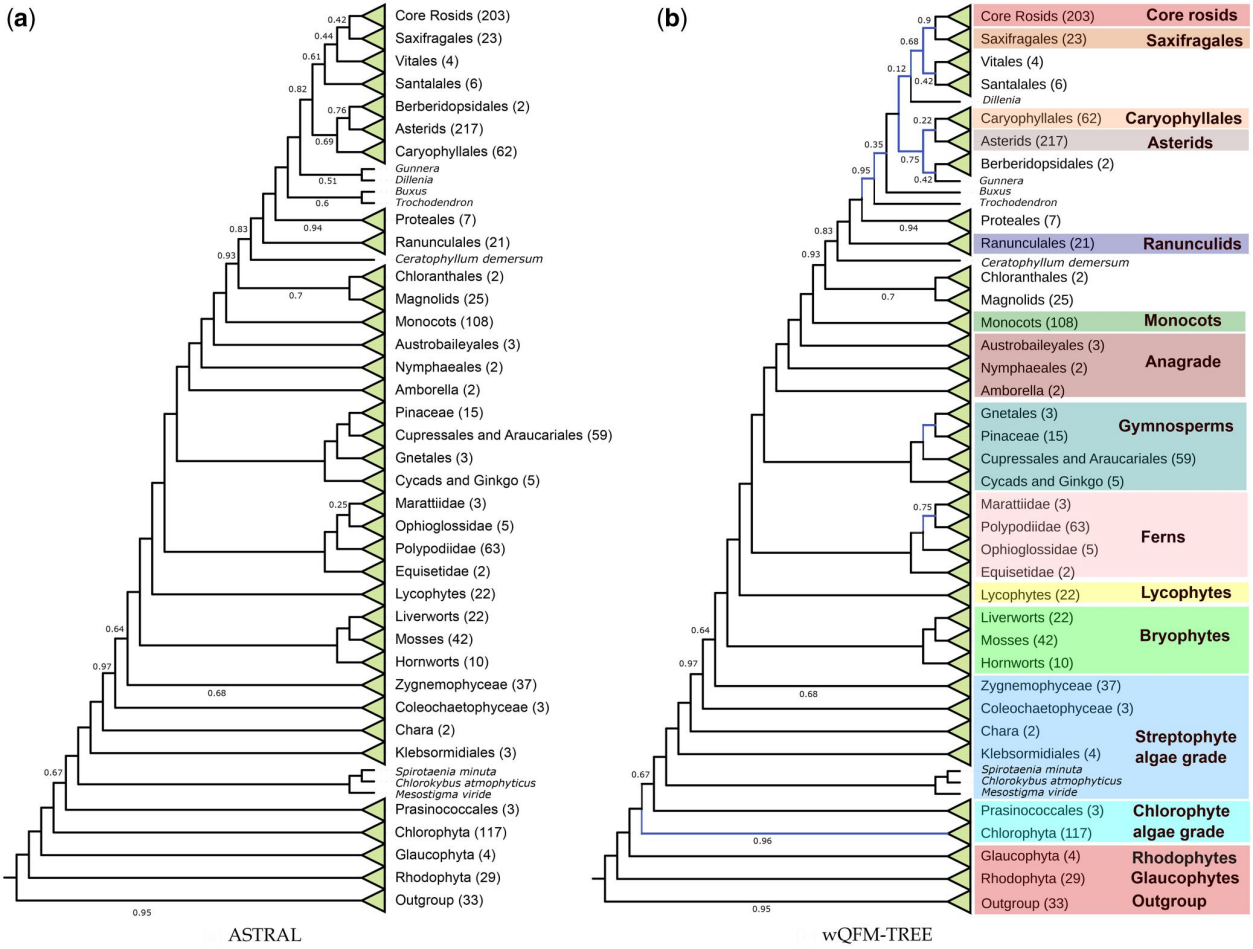
Phylogenetic trees reconstructed by wQFM-TREE using 410 single-copy nuclear gene families extracted from genome and transcriptome data from 1178 species, including green plants (Viridiplantae), glaucophytes, and red algae. Species numbers are shown for each lineage. The branches in the tree estimated by wQFM-TREE that differ from the ATRAL-estimated tree are shown in blue.

#### 4.1.2 Handling polytomies

wQFM-TREE can handle polytomies, making it suitable for partially resolved gene trees. Thus, wQFM-TREE can be applied even after removing low-support branches. However, similar to ASTRAL, the presence of polytomies increases the running time. In the presence of substantial amounts of polytomies, resolving them arbitrarily proves to be an effective strategy, resulting in a significant reduction in running time without sacrificing accuracy. As demonstrated in our study, resolving the polytomies does not have any notable impact on the species tree accuracy (Supplementary Fig. 4), while significantly improving the running time of wQFM-TREE (Supplementary Table 1).

### 4.2 Results on empirical dataset

We have re-analyzed the transcriptome dataset of 1178 species and 410 gene trees of plant species. The tree constructed by wQFM-TREE, shown in Fig. 4, is highly congruent with the tree estimated by ASTRAL-III v.5.7.8, which was presented in 1 kp (2019). wQFM-TREE successfully identified all the major clades, and the majority of the recovered relations between different clades are supported by other popular methods and do not violate any known consensus among scientists. Most clades produced by both algorithms are similar, with a few exceptions, including Core Rosids, Zygnematophyceae, and Rhodophyta. The RF distances between individual clades of the trees generated by wQFM-TREE and ASTRAL are provided in Supplementary Section 4.4. The key relationships are discussed below.

#### 4.2.1 Primary acquisition of plastid

**Figure 5.**
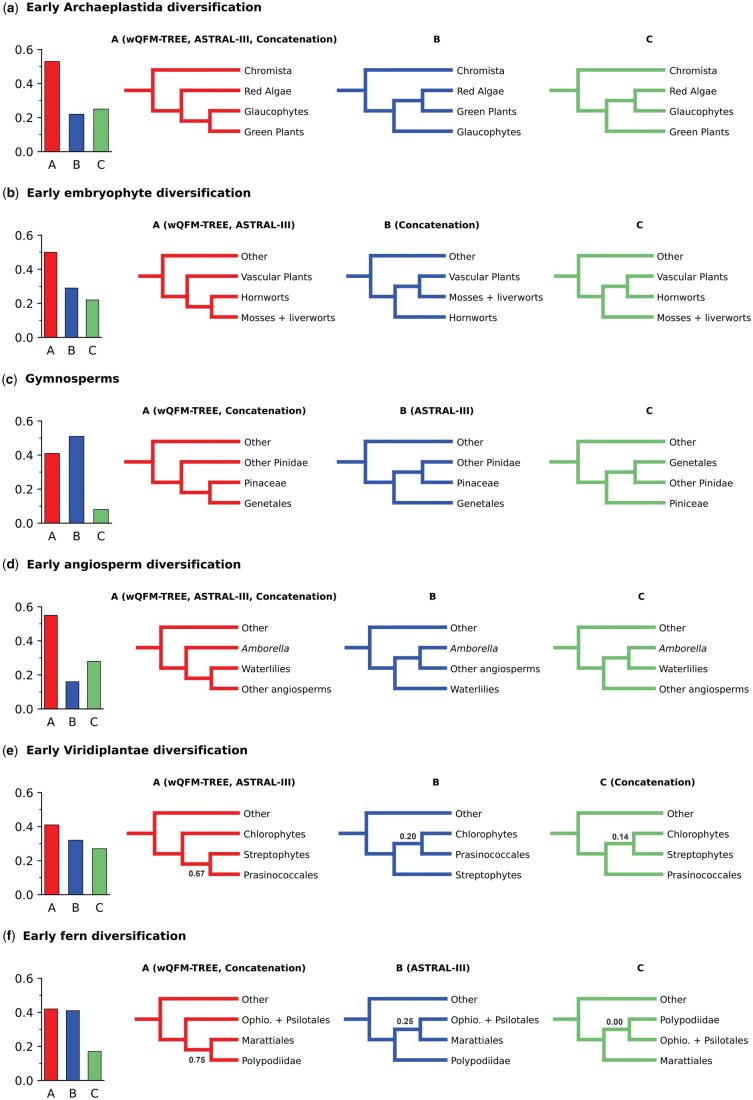
Gene-tree quartet frequencies (bar graphs) and local posterior probabilities (shown only when below 1.0) for alternative branching orders for contentious relationships in the plant phylogeny. (**a**) Early Archaeplastida diversification. (**b**) Early embryophyte diversification. (**c**) Gymnosperms. (**d**) Early angiosperm diversification. (**e**) Early Viridiplantae diversification. (**f**) Early fern diversification. Each of the cases contains four groups and three possible topologies. All the possible topologies with local posterior probabilities and quartet frequencies are shown, and the topologies recovered from wQFM-TREE, ASTRAL-III, and the concatenated supermatrix analysis are indicated.

Relationship among Viridiplantae, Glaucophyta, and Rhodophyta is crucial because it is related to the acquisition of plastid, which is a pivotal event in the history of life. The sister relationship of Viridiplantae and Glaucophyta found here (Fig. 5a), indicating that ancestral red algae lost flagella and peptidoglycan biosynthesis, is aligned with the ASTRAL-tree presented in 1 kp (2019).

#### 4.2.2 Viridiplantae

wQFM-TREE recovered the monophyletic relationship of Viridiplantae with early diverging Chlorophyta and Streptophyta which is consistent with prior studies. The placement of Prasinococcales is contentious and was found to be unstable in 1 kp (2019). However, the placement of Prasinococcales by wQFM-TREE is identical to the ASTRAL-estimated tree (Fig. 5e).

#### 4.2.3 Diversification within Chlorophyta and Streptophyta

**Figure 6.**
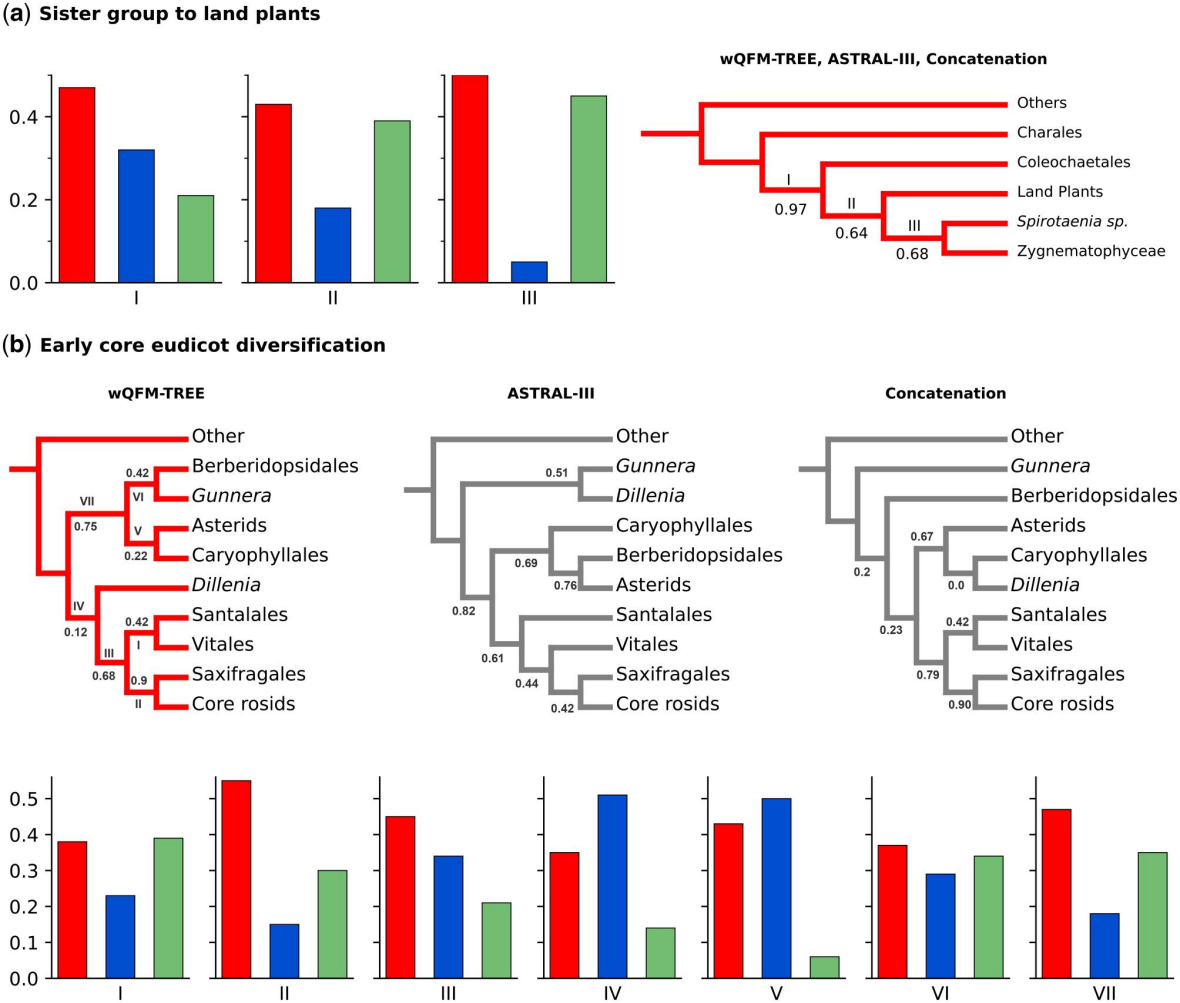
Gene-tree quartet frequencies and local posterior probabilities (shown only when below 1.0) for alternative branching orders for contentious relationships in the plant phylogeny. (**a**) The sister group to land plants. (**b**) Core Eudicot diversification. Here, each of the cases contains more than four groups. Therefore, the branches of the topology recovered from wQFM-TREE are labeled with Roman numerals, and the quartet frequencies of those branches are shown only for the wQFM-TREE topology. The topologies recovered from ASTRAL-III, and the concatenated supermatrix analysis are also indicated.

wQFM-TREE successfully reconstructed the monophyletic relationships within Trebouxiophyceae, Chlorophyceae, and Ulvophyceae, except Briopsidales (an order of green algae, in the class Ulvophyceae). Briopsidales did not exhibit monophyly with other Ulvophyceae; instead, it was positioned as a sister group to Pedinophyceae with a very low support (26.8%). ASTRAL also did not place Briopsidales as a sister to other Ulvophyceae. Trebouxiophyceae was placed as a sister to a clade containing Chlorophyceae and other Ulvophyceae. For Streptophyta, our analysis recovered *Mesostigma*, *Spirotaenia minuta*, and *Chlorokybus* within a clade that is sister to the remainder of Streptophyta, with the successive divergence of Klebsormidiales, Charophyceae, Coleochaetophyceae, and Zygnematophyceae relative to Embryophyta. These findings are consistent with the results of ASTRAL-III and the concatenated supermatrix analysis (Fig. 6a).

#### 4.2.4 Embryophyta

Although the relationships among the Bryophytes (mosses, liverworts, and hornworts) have been contentious and resolved as a grade in some studies, wQFM-TREE recovered Bryophytes as a monophyletic group similar to the ASTRAL-estimated tree and rejected the hypothesis that liverworts are sister to all other extant land plant lineages (Fig. 5b).

#### 4.2.5 Vascular plants

Lycophytes were correctly recovered as the sister group of ferns and seed plants. There are conflicting results between wQFM-TREE, supermatrix, and ASTRAL in the placement of Marattiales. wQFM-TREE placed it as a sister of Polypodiidae and ASTRAL placed it as a sister of Ophioglossidae. Both of the scenarios have nearly equal quartet support (Fig. 5f).

#### 4.2.6 Seed plants

Gymnosperms were correctly recovered as sister to flowering plants. In the case of gymnosperms, the placement of Gnetales conflicts strongly among different methods, which gives rise to the “Gnecup,” “Gnepine,” and “Gnetifer” hypotheses. From Fig. 5c, we observe that wQFM-TREE supported the “Gnepine” hypothesis and placed Gnetales as sisters to Pinales, which is in agreement with supermatrix analysis. However, it differed from ASTRAL, which supports the Gnetifer hypotheses, i.e. Gnetales is sister to Conifers (Araucariales, Cupressales, and Pinales) as a whole. The Gnepine and Gnetifer hypotheses have 41% and 51% quartet support, respectively (Fig. 5c).

In the case of Angiosperms, Amborellales, Nymphaeales, and Austrobaileyales were placed as successive sisters to all other angiosperms by all the methods. Chloranthales and Magnoliids were placed as sister groups but were resolved with poor support as successive sister lineages to all other Mesangiospermae (monocots, Ceratophyllum, and eudicots). Substantial gene-tree discordance was observed for relationships among Core Rosids, Saxifragales, Vitales, Dillenia, Santalales, Berberidopsidales, Caryophyllales, Asterids, and Gunnerales (Fig. 6b). We observe poor support in many branches of the trees produced by all the methods. Core Rosids, Saxifragales, Vitales, and Santalales were recovered as a monophyletic group across all the methods. However, the relative placements of these clades in the wQFM-TREE generated tree are aligned with the concatenated supermatrix analysis in which Vitales and Santalales were placed as sisters. ASTRAL placed Buxus and Trochodendron as sisters with low support (60% local posterior support). In contrast, wQFM-TREE placed Trochodendron as sister to Buxus and the core eudicots (Fig. 6b) with high support (95%), which is consistent with the results of the concatenated supermatrix analysis. There are additional differences (characterized by low supports) between wQFM-TREE and ASTRAL. For example, ASTRAL identified Gunnera and Dillenia as sisters, whereas wQFM-TREE placed Gunnera as a sister to Berberidopsidales, with both relationships having low supports.

### 4.3 Running time and memory consumption

We performed the experiments on a Linux machine with 64 GB RAM and Intel(R) Core (TM) i7-10700K 3.80 GHz processor. The runtimes and memory required by different methods on various datasets are presented in Supplementary Tables 6 and 7.

The running times of wQFM-TREE, ASTRAL, and TREE-QMC suggest that all of them are fast enough to handle large datasets containing thousands of taxa and genes. While their run times are comparable for relatively small numbers of taxa, both ASTRAL and TREE-QMC are faster than wQFM-TREE on larger datasets. However, wQFM-TREE remains fast enough to analyze the largest dataset analyzed in this study with 2000 taxa and 1000 genes in ∼6 h. We report the running time of wQFM-TREE on fully resolved gene trees, indicating that resolving polytomies substantially reduces runtime without sacrificing accuracy (Supplementary Fig. 4).

In order to demonstrate the scalability of wQFM-TREE on recent large phylogenomic datasets, wQFM-TREE was applied to the new extended avian dataset (Stiller *et al.* 2024) containing 363 species and 14 972 genes (exon loci). wQFM-TREE successfully analyzed this dataset within 11.6 h. The species tree estimated by wQFM-TREE is shown in Supplementary Fig. 5.

We observe that ASTRAL and wQFM-TREE have comparable memory usage, with both capable of analyzing up to 1000 taxa and 1000 gene trees within 7.6 GB of memory. In contrast, TREE-QMC demonstrates significantly greater memory efficiency, requiring only ∼295 MB of memory to handle the same dataset. wQFM-TREE required 33 GB of memory to analyze the avian dataset from Stiller *et al.* (2024).

## 5 Conclusions

In this work, we introduced wQFM-TREE, a novel method that allows the application of wQFM to gene trees without the need to decompose them into induced quartets. This innovation significantly enhances the scalability of wQFM, allowing it to handle datasets with thousands of taxa and genes efficiently. Our analyses, including the examination of the green plant dataset with over a thousand species, underscored the scalability of wQFM-TREE. Importantly, we have proposed several novel techniques, drawing from algorithms and combinatorics, for computing various quartet-based metrics (e.g. the number of satisfied or violated quartets) without explicitly enumerating the quartets in a given set of gene trees. These techniques not only enhance the scalability of wQFM but are also expected to facilitate quartet-based scoring techniques in general, eliminating the need to explicitly enumerate quartets in gene tree sets.

Although ASTRAL has been widely adopted, alternative heuristics like wQFM, despite being more accurate or comparable to ASTRAL (Mahbub *et al.* 2021, 2022), have faced challenges due to prohibitively long running times as the number of taxa increases. The techniques presented here address this scalability issue and make wQFM suitable to analyze very large datasets without sacrificing its accuracy. In this study, we demonstrate that wQFM-TREE is capable of analyzing large datasets comprising thousands of taxa and genes, consistently achieving accuracy comparable to or surpassing that of ASTRAL. Notably, on large simulated datasets with 500 and 1000 taxa, wQFM-TREE significantly outperformed ASTRAL across all six model conditions. Therefore, we believe that wQFM-TREE and the underlying novel techniques represent a significant advancement in the field of quartet-based phylogeny estimation.

wQFM-TREE is capable of analyzing thousands of taxa and genes. For instance, we successfully analyzed the 1KP dataset, containing 1178 taxa and 410 genes, in 5.5 h, and the avian dataset from Stiller *et al.* (2024), containing 363 species and 14,972 genes, in 11.6 h. Although at this stage, TREE-QMC is considerably faster than both ASTRAL and wQFM-TREE on large datasets, there is considerable potential for algorithmic improvements of wQFM-TREE, such as consolidating the input gene trees into a unified data structure to avoid redundant calculations, employing heuristic strategies to reduce the number of FM iterations, etc. The partitioning technique of the QFM algorithm is inherently parallelizable, and future studies need to develop a parallel version of wQFM-TREE. The scalability of wQFM-TREE can be further enhanced by the proposed divide-and-conquer framework to speed up summary methods (Bayzid *et al.* 2014). wQFM-TREE can handle single-copy rooted and unrooted gene trees. Extending it for multi-copy gene trees would be an interesting future research direction.

## Supplementary Material

vbaf053_Supplementary_Data

## Data Availability

wQFM-TREE is freely available in open source form at https://github.com/abdur-rafi/wQFM-TREE. All the datasets analyzed in this paper are from previously published studies and are publicly available.
