# Supplementary material for: wQFM-TREE: highly accurate and scalable quartet-based species tree inference from gene trees
Source: Bioinform Adv. 2025 Mar 13;5(1):vbaf053. doi: 10.1093/bioadv/vbaf053 (PMC11932941; doi:10.1093/bioadv/vbaf053)
Supplement: vbaf053_Supplementary_Data [file vbaf053_supplementary_data.pdf]

Supplementary Material to

wQFM-TREE: highly accurate and scalable quartet-based species tree inference from gene trees

Abdur Rafi[1,†], Ahmed Mahir Sultan Rumi[1,†], Sheikh Azizul Hakim[1], Sohaib[1], Md. Toki Tahmid[1], Rabib Jahin Ibn Momin[1], Tanjeem Azwad Zaman[1], Rezwana Reaz[1], and Md. Shamsuzzoha Bayzid[1,*]

[1]Department of Computer Science and Engineering
Bangladesh University of Engineering and Technology
Dhaka-1205, Bangladesh
[†]These authors contributed equally to this work
[*]Corresponding author: shams_bayzid@cse.buet.ac.bd

Contents

1 Overview of wQFM and wQFM-TREE **3**

2 Additional details about score computation **6**
2.1 Additional details about weight computation 7
2.1.1 Computing $w(PA_{i,j}^{(g,u)})$ and $w(PB_{i,j}^{(g,u)})$ 7
2.1.2 Computing $w(PB_{k}^{(g,u)})$ 7
2.1.3 Efficient formulation of $w(S^{(g,u)})$ 8
2.1.4 Computing $w(PA^{(g)})$ and $w(PB^{(g)})$ 8
2.1.5 Efficient formulation of $w(U^{(g,u)})$ 8
2.2 Example demonstrating the score computation method 9
2.2.1 Computing $w(S^{(g)})$ 10
2.2.2 Computing $w\left(S^{(g)} \cup V^{(g)} \cup U^{(g)}\right)$ 12
2.2.3 Computing $w(U^{(g)})$ 12
2.3 Weight normalization 14
2.4 Gain calculation 14

3 Time complexity **15**
3.1 Time complexity for fully resolved gene trees with the assumption of balanced partitioning 16
3.2 Time complexity without the assumptions 16

4 Additional results **16**
4.1 Comparing wQFM-TREE with wQFM 16
4.2 Handling polytomies 18
4.3 Results of statistical tests 19
4.4 RF distances between clades in the wQFM-TREE and ASTRAL results for the plant dataset 20
4.5 Running time and memory consumption 21
4.6 Results on the avian dataset 22

List of Tables

1 Results of statistical significance tests (p-values) between wQFM-TREE and ASTRAL-III, and wQFM-TREE and TREE-QMC for the 200-taxon dataset (see Figure 2 in the main text). 19
2 Results of statistical significance tests (p-values) between wQFM-TREE and ASTRAL-III, and wQFM-TREE and TREE-QMC for the analysis with varying number of taxa (see Figure 3 in the main text). 19
3 RF distances between individual clades of wQFM-TREE and ASTRAL-III with four or more taxa. 20
4 Runtime of various methods on the datasets analyzed in this study. . . . 21
5 Memory consumption of wQFM-TREE, ASTRAL-III, and TREE-QMC on some of the datasets analyzed in this study. 21

List of Figures

1 Divide and conquer approach of wQFM 3
2 An example gene tree to demonstrate the score calculation process 9
3 Comparison of wQFM and wQFM-TREE on 37-taxon mammalian and 48-taxon avian simulated datasets. 17
4 Performance of wQFM-TREE with and without resolving polytomies. . . 18
5 Phylogenetic tree reconstructed by wQFM-TREE on the avian dataset from Stiller et al. [2024]. 22

These supplementary materials present additional details about the proposed technique for computing scores of candidate bipartitions from gene trees, the time complexity of wQFM-TREE, and additional results, figures, and tables.

1 Overview of wQFM and wQFM-TREE

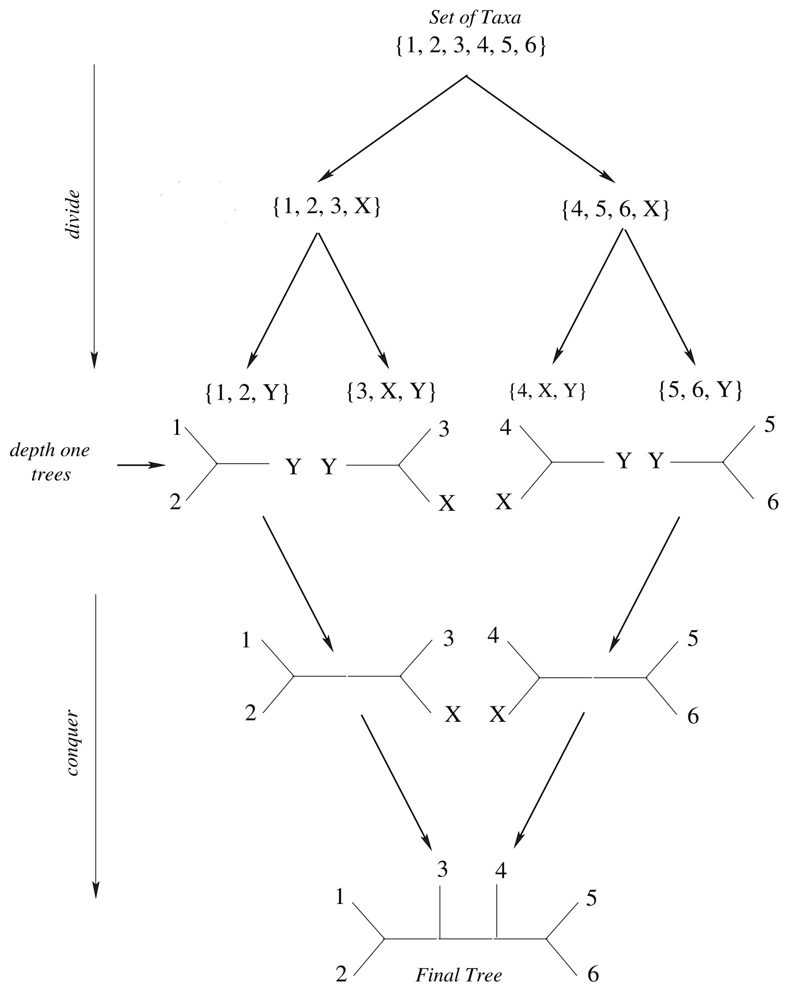

Figure 1: **Divide and conquer approach of wQFM**. *Divide:* At each step, the input set of taxa of this step is partitioned into two sets and a unique dummy taxon is added to both of the sets. These sets are inputs to the successive divide steps. If at any step, the size of the taxa set becomes less than or equal to three, a depth one tree over the taxa set is returned. *Conquer:* At each step, there are two trees corresponding to the divide calls initiated at this step. These two trees are joined on the dummy taxon introduced at this step during divide. For example, the leftmost two depth one trees, when returned to its caller, are joined on the dummy taxon Y.

wQFM-TREE follows exactly the same divide-and-conquer approach as wQFM. However, the underlying techniques in each divide step is different as wQFM-TREE does all the computations directly from the input gene trees. Here, an initial bipartition is created at each divide step using a greedy consensus tree of the gene trees following **Algorithm 1**. Then, the the initial bipartition is modified and refined through the FM algorithm Fiduccia and Mattheyses [1982] which uses the scoring process presented in **Algorithm 2**.

Algorithm 1: Creation of initial bipartition for a taxa set which will be further refined by the FM algorihtm

---

```
Input  : 𝒢, set of gene trees
         S, set of taxa of a subproblem (may contain dummy taxa)
Output: (S_A, S_B) : A bipartition over the set S

𝒞 ← ConstructGreedyConsensusTree(𝒢)
S_A ← ∅
S_B ← ∅
maxscore ← 0
foreach edge e ∈ 𝒞 do
    (A, B) ← bipartition defined by the edge e
    /* S may contain dummy taxa and therefore (A, B) directly is not a
       candidate for initial bipartition, we create candidate initial
       bipartition by further processing                              */
    C_A ← ∅
    C_B ← ∅
    foreach t in S do
        if t ∈ A then C_A ← C_A ∪ {t} ; // real taxa present in A
        else if t ∈ B then C_B ← C_B ∪ {t} ; // real taxa present in B
        else // t is a dummy taxa
            W_A ← weighted sum of real taxa "under taxon t" present in A
            W_B ← weighted sum of real taxa "under taxon t" present in B
            if W_A ≥ W_B then C_A ← C_A ∪ {t} ;
            else C_B ← C_B ∪ {t} ;
        end
    end
    candidatescore ← Score(C_A, C_B,G)
    if candidatescore > maxscore then
        maxscore ← candidatescore
        S_A ← C_A, S_B ← C_B
    end
end
return (S_A, S_B)
```

Algorithm 2: Scoring a bipartition (used by FM algorithm to find the best biparition)

Input : $\mathcal{G}$, set of gene trees
(S_A, S_B) A bipartition of taxon set
Output: Bipartition Score of (S_A, S_B)

```
score ← 0
foreach gene tree g ∈ 𝒢 do
    w_svu ← sum of weights of all quartets(resolved or unresolved) that are
     satisfied or violated by the bipartition (S_A, S_B) ;  // Detailed formulation
     in Section 2.5.2 in main text
    w_sg ← 0, w_ug ← 0
    foreach node u in g do
        /* sum of weights of the quartet set S^(g,u) defined and formulated in
           detail in Section 2.5.1 in main text                              */
        w_sgu ← sum of weights of satisfied quartets with anchor at node u
        /* sum of weights of the quartet set U^(g,u) defined and formulated in
           detail in Section 2.5.3 in main text                              */
        w_ugu ← sum of weights of unresolved satisfied or violated quartets at
          node u
        w_sg ← w_sg + w_sgu
        w_ug ← w_ug + w_ugu
    end
    score ← score + 2 · w_sg − w_svu + w_ug ;       // Derivation in Section 2.5
end
return score
```

2 Additional details about score computation

We revisit some terminologies for the following subsections. Let $\mathcal{G}$ be a set of unrooted gene trees on taxa set $\mathcal{X}$. We allow the gene trees to be non-binary and incomplete (i.e., some taxa could be missing in a gene tree). Let $\mathcal{X}^{(g)}$ be the set of real taxa in a gene tree $g \in \mathcal{G}$. Let (A, B) be the given bipartition for which we want to calculate the score for our FM algorithm. Let R_A and D_A be the sets of real and dummy taxa in A, respectively. Let F_A be the set of all real taxa that are either in R_A or under a dummy taxon $X \in D_A$, i.e., $F_A = (\bigcup_{X \in D_A} X_R) \cup R_A$. We define R_B, D_B, F_B similarly.

Our algorithm assigns weight to each real taxon in $\mathcal{X}$ according to Equation 1 in the main paper. These weights are used to calculate the weights of quartets. Let $a, b, c, d \in \mathcal{X}$. We define the weight of an unordered pair of taxa, $p = \{a, b\}$ as $w(p) = w(a) \cdot w(b)$. We consider a quartet $q = ab|cd$ to be composed of 2 unordered pairs $\{a, b\}$ and $\{c, d\}$. We define its weight $w(ab|cd)$ to be $w(\{a, b\}) \cdot w(\{c, d\})$. We define the weight of a set Q of quartets as $w(Q) = \sum_{q \in Q} w(q)$. We define the weight of a set of unordered pairs similarly.

Lemma 1. *Let $S^{(g,u)}$ be the subset of all satisfied quartets $ab|cd$ with respect to bipartition (A, B) (a, b in parition A and c, d in partition B) in gene tree g which have u as a quartet anchor directly connected to a, b in the restricted quartet tree. Then $S^{(g,u)}$ for all internal nodes u of gene tree g form a partition of the set $S^{(g)}$ of satisfied resolved quartets of gene tree g.*

Proof. To prove the statement, we need to show that the union of $S^{(g,u)}$ over all internal nodes u is $S^{(g)}$ and any pair of $S^{(g,u)}$ and $S^{(g,v)}$, for two distinct nodes u and v, are disjoint.

First, we show that the union of $S^{(g,u)}$ over all internal nodes u is $S^{(g)}$. We consider a satisfied (resolved) quartet $ab|cd$ where $a, b \in F_A$; $c, d \in F_B$. Each quartet of leaves in the gene tree defines two anchor nodes. For the quartet $ab|cd$, one anchor node is directly connected to a, b and the other to c, d in the quartet tree. Let z be the internal node, which is the anchor directly connected to a, b. Of course, a and b belong to two distinct components in $C^{(g,z)}$, and both c, d pertain to a third component of $C^{(g,z)}$. Thus, every quartet belongs to $S^{(g,u)}$ for at least one internal node u. Thus, the union of $S^{(g,u)}$ over all internal nodes u is $S^{(g)}$.

Now, we show that any pair of $S^{(g,u)}$ and $S^{(g,v)}$, for two distinct nodes u and v, are disjoint. Again, we consider a satisfied (resolved) quartet $ab|cd$ where $a, b \in F_A$; $c, d \in F_B$ and let z be the internal node, which is the anchor directly connected to a, b. We consider the path from a to b in gene tree g. For any internal node x outside of the path, a and b are in the same component element of $C^{(g,x)}$, which violates the definition of $S^{(g,x)}$. For any node x in the path except z, nodes a, c, d or b, c, d will be in the same component element of $C^{(g,x)}$. Therefore, the quartet $ab|cd$ belongs to only $S^{(g,z)}$. Therefore, a quartet $ab|cd$ can only belong to $S^{(g,u)}$ for one internal node u which is the anchor node defined in the gene tree by the leaves a, b, c, d and directly connected to a, b in the quartet tree. Thus, any pair of $S^{(g,u)}$ and $S^{(g,v)}$, for two different nodes u and v, are disjoint. This completes our proof.

□

2.1 Additional details about weight computation

Now, let $F_A^{(g,u,i)}$ be the set of real taxa of F_A that are in $C_i^{(g,u)}$, $X_R^{(g,u,i)}$ be the set of real taxa in $C_i^{(g,u)}$ that are under a dummy taxon X and $R_A^{(g,u,i)}$ be the set of real taxa of R_A that are in $C_i^{(g,u)}$. We similarly define $F_B^{(g,u,i)}$ and $R_B^{(g,u,i)}$.

2.1.1 Computing $w(PA_{i,j}^{(g,u)})$ and $w(PB_{i,j}^{(g,u)})$

Let $P_1 = \{\{a,b\} : a \in F_A^{(g,u,i)}, b \in F_A^{(g,u,j)}\}$. Here P_1 contains all elements of $PA_{i,j}^{(g,u)}$. However, it also contains the unordered pairs $\{a,b\}$ where both a and b are under the same dummy taxon. Let $P_2 = \bigcup_{X \in D_A} \{\{a,b\} : a \in X_R^{(g,u,i)}, b \in X_R^{(g,u,j)}\}$. It contains all pairs $\{a,b\}$ where $a \in F_A^{(g,u,i)}$, $b \in F_A^{(g,u,j)}$ and both a and b are under the same dummy taxon. Now, P_2 is a subset of P_1 and $PA_{i,j}^{(g,u)} = P \backslash Q$. Thus, $w(PA_{i,j}^{(g,u)}) = w(P_1) - w(P_2)$.

Now $w(P_1) = w(F_A^{(g,u,i)})w(F_A^{(g,u,j)})$ and $w(P_2) = \sum_{X \in D_A} w(X_R^{(g,u,i)})w(X_R^{(g,u,j)})$. Thus, we obtain the formula of $w(PA_{i,j}^{(g,u)})$ as follows.

$$w(PA_{i,j}^{(g,u)}) = w(F_A^{(g,u,i)})w(F_A^{(g,u,j)}) - \sum_{X \in D_A} w(X_R^{(g,u,i)})w(X_R^{(g,u,j)})$$

We can compute $w(F_A^{(g,u,i)})$ and $w(X_R^{(g,u,i)})$ for all internal nodes u of gene tree g through a single post-order traversal of g after rooting it arbitrarily. Similarly,

$$w(PB_{i,j}^{(g,u)}) = w(F_B^{(g,u,i)})w(F_B^{(g,u,j)}) - \sum_{X \in D_B} w(X_R^{(g,u,i)})w(X_R^{(g,u,j)})$$

2.1.2 Computing $w(PB_k^{(g,u)})$

To compute $w(PB_k^{(g,u)})$, we can follow a similar approach to that we used for $w(PB_{i,j}^{(g,u)})$ with some minor modifications.

$$\begin{aligned} w(PB_k^{(g,u)}) &= \frac{1}{2}(w(F_B^{(g,u,k)})w(F_B^{(g,u,k)}) - w(R_B^{(g,u,k)}) - \sum_{X \in D_B} w(X_R^{(g,u,k)})w(X_R^{(g,u,k)})) \\ &= \frac{1}{2}(w(F_B^{(g,u,k)})^2 - w(R_B^{(g,u,k)}) - \sum_{X \in D_B} w(X_R^{(g,u,k)})^2) \end{aligned}$$

This expression differs from that of $w(PB_{i,j}^{(g,u)})$ for a few reasons. Unlike the case of $w(PB_{i,j}^{(g,u)})$, $\left(w(F_B^{(g,u,k)})\right)^2$ also contains the sum of weights of $\{a,a\}$, where $a \in F_B^{(g,u,k)}$. We do not include $\{a,a\}$ by definition in $PB_k^{(g,u)}$ as a real taxon cannot participate twice in a quartet. The weights of $\{a,a\}$ where $a \in X_R$ for some $X \in D_B$ are already included in $\sum_{X \in D_B}\left(w(X_R^{(g,u,k)})\right)^2$. Therefore, we need to subtract the weights of $\{a,a\}$, where a is not under any dummy taxon, that is, $a \in R_B^{(g,u,k)}$. As $w(a) = 1$ for $a \in R_B^{(g,u,k)}$, the sum of the weights of these $\{a,a\}$ is given by $w(R_B^{(g,u,k)})$. Moreover, since both taxa are from the same set, the weight of all unordered pairs is considered twice. Hence, by dividing the expression by 2, we obtain the final formula.

2.1.3 Efficient formulation of $w(S^{(g,u)})$

We compute $w(S^{(g,u)})$ efficiently in the following way. Let $w(PB^{(g,u)}) = \sum_k w(PB_k^{(g,u)})$. For a certain i, j,

$$\begin{aligned}\sum_{k \neq i,j} w(S_{i,j,k}^{(g,u)}) &= \sum_{k \neq i,j} w(PA_{i,j}^{(g,u)}) w(PB_k^{(g,u)}) \\ &= w(PA_{i,j}^{(g,u)}) \sum_{k \neq i,j} w(PB_k^{(g,u)}) \\ &= w(PA_{i,j}^{(g,u)}) \cdot (w(PB^{(g,u)}) - w(PB_i^{(g,u)}) - w(PB_j^{(g,u)}))\end{aligned}$$

Thus, we get

$$\begin{aligned}w(S^{(g,u)}) &= \sum_{i<j} \sum_{k \neq i,j} w(S_{i,j,k}^{(g,u)}) \\ &= \sum_{i<j} w(PA_{i,j}^{(g,u)}) \cdot (w(PB^{(g,u)}) - w(PB_i^{(g,u)}) - w(PB_j^{(g,u)}))\end{aligned}$$

2.1.4 Computing $w(PA^{(g)})$ and $w(PB^{(g)})$

Similar to 2.1.2, $PA^{(g)}$ contains pairs $\{a, b\}$ where both a, b are from the same set $\mathcal{X}^{(g)} \cap F_A$. Therefore,

$$w(PA^{(g)}) = \frac{1}{2}(w(F_A \cap \mathcal{X}^{(g)})^2 - w(R_A \cap \mathcal{X}^{(g)}) - \sum_{X \in D_A} w(X_R \cap \mathcal{X}^{(g)})^2)$$

Similarly,

$$w(PB^{(g)}) = \frac{1}{2}(w(F_B \cap \mathcal{X}^{(g)})^2 - w(R_B \cap \mathcal{X}^{(g)}) - \sum_{X \in D_B} w(X_R \cap \mathcal{X}^{(g)})^2)$$

2.1.5 Efficient formulation of $w(U^{(g,u)})$

$$w(U^{(g,u)}) = \sum_{i<j} w(PA_{i,j}^{(g,u)})(\sum_{k<l;k,l \notin \{i,j\}} w(PB_{k,l}^{(g,u)}))$$

Let $SB_i^{(g,u)} = \sum_{k<l;(k=i) \vee (l=i)} w(PB_{k,l}^{(g,u)})$ and $SB^{(g,u)} = \sum_{k<l} w(PB_{k,l}^{(g,u)})$. Using these definitions, we can refine the expression as follows.

$$\sum_{k<l;k,l \notin \{i,j\}} w(PB_{k,l}^{(g,u)}) = SB^{(g,u)} - SB_i^{(g,u)} - SB_j^{(g,u)} + w(PB_{i,j}^{(g,u)})$$

Thus, we obtain the following equation.

$$\begin{aligned}w(U^{(g,u)}) &= \sum_{i<j} w(PA_{i,j}^{(g,u)})(\sum_{k<l;k,l \notin \{i,j\}} w(PB_{k,l}^{(g,u)})) \\ &= \sum_{i<j} w(PA_{i,j}^{(g,u)})(SB^{(g,u)} - SB_i^{(g,u)} - SB_j^{(g,u)} + w(PB_{i,j}^{(g,u)}))\end{aligned}$$

2.2 Example demonstrating the score computation method

We demonstrate the score calculation process by calculating the score of the bipartition $(A, B) = (\{1, 4, 12, X\}, \{2, 6, 7, Y\})$ for the unrooted gene tree g shown in Figure 2a. Here, X and Y are dummy taxa. Their tree structures are shown in Figure 2a. From the tree structure, $X_R = \{5, 10, 11\}$ and $Y_R = \{3, 8, 9\}$. The weights of all the real taxa which are not under X or Y (taxa 1, 2, 4, 6, 7, 12) are 1. Using the weighting mechanism described in Section 2.3 of the main paper, we calculate the taxa weights in X_R and Y_R.

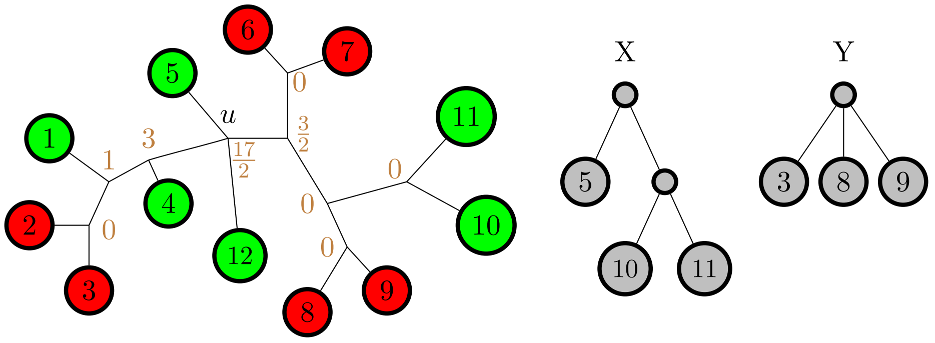

(a) Example gene tree and the tree structure of the dummy taxa X and Y. The values at each internal node of the gene tree denote the value of $w(S^{(g,u)})$ for that node.

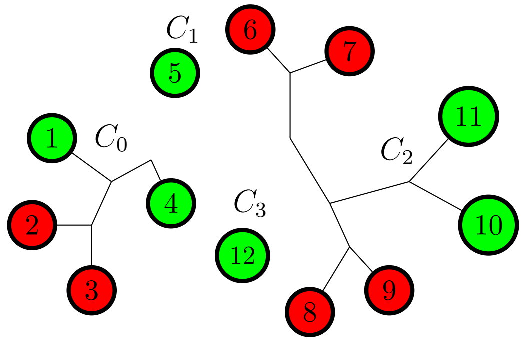

(b) Resulting components after removing the internal node u. The components are labeled $C_0 - C_3$ in an arbitrary order.

Figure 2: An example gene tree to demonstrate our score calculation process for the bipartition $(\{1, 4, 12, X\}, \{2, 6, 7, Y\})$.

For taxa in X_R, $w(5) = \frac{1}{2}$, $w(10) = \frac{1}{4}$, and $w(11) = \frac{1}{4}$. For taxa in Y_R, $w(3) = \frac{1}{3}$, $w(8) = \frac{1}{3}$, and $w(9) = \frac{1}{3}$.

From A and B, $R_A = \{1, 4, 12\}$, $D_A = \{X\}$, $F_A = \{1, 4, 5, 10, 11, 12\}$, $R_B = \{2, 6, 7\}$,$D_B = \{Y\}$, $F_B = \{2, 3, 6, 7, 8, 9\}$.

We recall that,

$$Score(A, B, \mathcal{G}) = \sum_{g \in \mathcal{G}} (2w(S^{(g)}) - w(S^{(g)} \cup V^{(g)} \cup U^{(g)}) + w(U^{(g)}))$$

Here, $\mathcal{G}$ is a set of unrooted gene trees. We have one gene tree g in $\mathcal{G}$ in our example. Therefore, the equation becomes,

$$Score(A, B, \mathcal{G}) = 2w(S^{(g)}) - w(S^{(g)} \cup V^{(g)} \cup U^{(g)}) + w(U^{(g)})$$

We show the detailed calculation of $w(S^{(g,u)})$ and $w(U^{(g,u)})$ only for the internal node u, where u is the marked node in Figure 2a. Removing u creates 4 components, $C_0^{(g,u)}$, $C_1^{(g,u)}$, $C_2^{(g,u)}$, $C_3^{(g,u)}$. These components are shown in Figure 2b, labeled by C_0, C_1, C_2, C_3.

2.2.1 Computing $w(S^{(g)})$

We determine $w(S^{(g)}) = \sum_{v \in g} w(S^{(g,v)})$. We present the detailed computation of $w(S^{(g,u)})$. We recall that,

$$\begin{aligned} w(S^{(g,u)}) &= \sum_{i<j} \sum_{k \neq i,j} w(S_{i,j,k}^{(g,u)}) \\ &= \sum_{i<j} w(PA_{i,j}^{(g,u)}) \cdot (w(PB^{(g,u)}) - w(PB_i^{(g,u)}) - w(PB_j^{(g,u)})) \end{aligned}$$

To compute $w(S^{(g,u)})$, we need to determine $w(PA_{i,j}^{(g,u)})$ for each $i < j$ and $w(PB_i^{(g,u)})$ for each i . For this task, We calculate the value of all the required expressions as shown below.

$F_A^{(g,u,0)} = \{1, 4\}$, $F_A^{(g,u,1)} = \{5\}$, $F_A^{(g,u,2)} = \{10, 11\}$, $F_A^{(g,u,3)} = \{12\}$.

$w(F_A^{(g,u,0)}) = 2$, $w(F_A^{(g,u,1)}) = \frac{1}{2}$, $w(F_A^{(g,u,2)}) = \frac{1}{2}$, $w(F_A^{(g,u,3)}) = 1$.

$F_B^{(g,u,0)} = \{2, 3\}$, $F_B^{(g,u,1)} = \{\}$, $F_B^{(g,u,2)} = \{6, 7, 8, 9\}$, $F_B^{(g,u,3)} = \{\}$.

$w(F_B^{(g,u,0)}) = \frac{4}{3}$, $w(F_B^{(g,u,1)}) = 0$, $w(F_B^{(g,u,2)}) = \frac{8}{3}$, $w(F_B^{(g,u,3)}) = 0$.

$R_A^{(g,u,0)} = \{1, 4\}$, $R_A^{(g,u,1)} = \{\}$, $R_A^{(g,u,2)} = \{\}$, $R_A^{(g,u,3)} = \{12\}$.

$w(R_A^{(g,u,0)}) = 2$, $w(R_A^{(g,u,1)}) = 0$, $w(R_A^{(g,u,2)}) = 0$, $w(R_A^{(g,u,3)}) = 1$.

$R_B^{(g,u,0)} = \{2\}$, $R_B^{(g,u,1)} = \{\}$, $R_B^{(g,u,2)} = \{6, 7\}$, $R_B^{(g,u,3)} = \{\}$

$w(R_B^{(g,u,0)}) = 1$, $w(R_B^{(g,u,1)}) = 0$, $w(R_B^{(g,u,2)}) = 2$, $w(R_B^{(g,u,3)}) = 0$.

$X_R^{(g,u,0)} = \{\}$, $X_R^{(g,u,1)} = \{5\}$, $X_R^{(g,u,2)} = \{10, 11\}$, $X_R^{(g,u,3)} = \{\}$.

$w(X_R^{(g,u,0)}) = 0$, $w(X_R^{(g,u,1)}) = \frac{1}{2}$, $w(X_R^{(g,u,2)}) = \frac{1}{2}$, $w(X_R^{(g,u,3)}) = 0$.

$Y_R^{(g,u,0)} = \{3\}$, $Y_R^{(g,u,1)} = \{\}$, $Y_R^{(g,u,2)} = \{8, 9\}$, $Y_R^{(g,u,3)} = \{\}$.

$w(Y_R^{(g,u,0)}) = \frac{1}{3}$, $w(Y_R^{(g,u,1)}) = 0$, $w(Y_R^{(g,u,2)}) = \frac{2}{3}$, $w(Y_R^{(g,u,3)}) = 0$.

Now, we can compute $w(PA_{i,j}^{(g,u)})$ for each $i < j$

$$w(PA_{0,1}^{(g,u)}) = w(F_A^{(g,u,0)})w(F_A^{(g,u,1)}) - w(X_R^{(g,u,0)})w(X_R^{(g,u,1)}) = 2 \cdot \frac{1}{2} - 0 \cdot \frac{1}{2} = 1.$$

Similarly,

$$w(PA_{0,2}^{(g,u)}) = 1, w(PA_{0,3}^{(g,u)}) = 2, w(PA_{1,2}^{(g,u)}) = 0, w(PA_{1,3}^{(g,u)}) = \frac{1}{2}, w(PA_{2,3}^{(g,u)}) = \frac{1}{2}.$$

Then, we compute $w(PB_k^{(g,u)})$ for each k.

$$w(PB_0^{(g,u)}) = \frac{1}{2}(w(F_B^{(g,u,0)})^2 - w(R_B^{(g,u,0)}) - w(Y_R^{(g,u,0)})^2) = \frac{1}{2}((\frac{4}{3})^2 - 1 - \frac{1}{3^2}) = \frac{1}{3}.$$

Similarly,

$$w(PB_1^{(g,u)}) = 0, w(PB_2^{(g,u)}) = \frac{7}{3}, w(PB_3^{(g,u)}) = 0.$$

Then, we compute the value of $PB^{(g,u)}$.

$$PB^{(g,u)} = w(PB_0^{(g,u)}) + w(PB_1^{(g,u)}) + w(PB_2^{(g,u)}) + w(PB_3^{(g,u)}) = \frac{8}{3}$$

We are done computing all the necessary values required to compute $\sum_{k\neq i,j} w(S_{i,j,k}^{(g,u)})$ for a fixed i, j.

$$\sum_{k\neq i,j} w(S_{i,j,k}^{(g,u)}) = w(PA_{i,j}^{(g,u)}) \cdot (w(PB^{(g,u)}) - w(PB_i^{(g,u)}) - w(PB_j^{(g,u)}))$$

$$\begin{aligned}\sum_{k\neq 0,1} w(S_{0,1,k}^{(g,u)}) &= w(PA_{0,1}^{(g,u)}) \cdot (w(PB^{(g,u)}) - w(PB_0^{(g,u)}) - w(PB_1^{(g,u)})) \\ &= 1 \cdot (\frac{8}{3} - \frac{1}{3} - 0) = \frac{7}{3}\end{aligned}$$

Similarly,

$$\sum_{k\neq 0,2} w(S_{0,2,k}^{(g,u)}) = 0, \sum_{k\neq 0,3} w(S_{0,3,k}^{(g,u)}) = \frac{14}{3}, \sum_{k\neq 1,2} w(S_{1,2,k}^{(g,u)}) = 0,$$
$$\sum_{k\neq 1,3} w(S_{1,3,k}^{(g,u)}) = \frac{4}{3}, \sum_{k\neq 2,3} w(S_{2,3,k}^{(g,u)}) = \frac{1}{6},$$

Finally, we compute $w(S^{(g,u)})$.

$$\begin{aligned}w(S^{(g,u)}) =& \sum_{i<j}\sum_{k\neq i,j} S_{i,j,k}^{(g,u)} \\ =& \sum_{k\neq 0,1} w(S_{0,2,k}^{(g,u)}) + \sum_{k\neq 0,2} w(S_{0,2,k}^{(g,u)}) + \sum_{k\neq 0,3} w(S_{0,3,k}^{(g,u)}) + \sum_{k\neq 1,2} w(S_{1,2,k}^{(g,u)}) \\ &+ \sum_{k\neq 1,3} w(S_{1,3,k}^{(g,u)}) + \sum_{k\neq 2,3} w(S_{2,3,k}^{(g,u)}) \\ =& \frac{7}{3} + 0 + \frac{14}{3} + 0 + \frac{4}{3} + \frac{1}{6} = \frac{17}{2}\end{aligned}$$

By performing similar calculations for the other nodes, we obtain the value of $w(S^{(g)})$.

$$\begin{aligned} w(S^{(g)}) &= \sum_{u\in g} w(S^{(g,u)}) \\ &= 0 + 1 + 3 + \frac{17}{2} + 0 + \frac{3}{2} + 0 + 0 + 0 = 14 \end{aligned}$$

2.2.2 Computing $w\left(S^{(g)} \cup V^{(g)} \cup U^{(g)}\right)$

Now, we compute $w(S^{(g)} \cup V^{(g)} \cup U^{(g)})$. We need to compute $w(PA^{(g)})$ and $w(PA^{(g)})$ for this task.

Since we have no missing taxon, $\mathcal{X}^{(g)} = \mathcal{X}$, $F_A \cap \mathcal{X}^{(g)} = F_A$, $F_B \cap \mathcal{X}^{(g)} = F_B$, $R_A \cap \mathcal{X}^{(g)} = R_A$, $R_B \cap \mathcal{X}^{(g)} = R_B$, $X_R \cap \mathcal{X}^{(g)} = X_R$, and $Y_R \cap \mathcal{X}^{(g)} = Y_R$.

$$\begin{aligned} &w(F_A \cap \mathcal{X}^{(g)}) = 4, w(F_B \cap \mathcal{X}^{(g)}) = 4, w(R_A \cap \mathcal{X}^{(g)}) = 3 \\ &w(R_B \cap \mathcal{X}^{(g)}) = 3, w(X_R \cap \mathcal{X}^{(g)}) = 1, w(Y_R \cap \mathcal{X}^{(g)}) = 1 \end{aligned}$$

Hence,

$$\begin{aligned} w(PA^{(g)}) &= \frac{1}{2}(w(F_A \cap \mathcal{X}^{(g)})^2 - w(R_A \cap \mathcal{X}^{(g)}) - w(X_R \cap \mathcal{X}^{(g)})^2) \\ &= \frac{1}{2}(16 - 3 - 1) = 6 \end{aligned}$$

Similarly, $w(PB^{(g)}) = 6$. Finally, we compute

$$w(S^{(g)} \cup V^{(g)} \cup U^{(g)}) = w(PA^{(g)}) \cdot w(PB^{(g)}) = 6 \cdot 6 = 36$$

2.2.3 Computing $w(U^{(g)})$

The only task remaining is to compute $w(U^{(g)})$. As u is the only polytomy node, $w(U^{(g)}) = w(U^{(g,u)})$. Therefore, we only need to compute $w(U^{(g,u)})$. For this task, we compute $w(PB_{i,j}^{(g,u)})$ for all $i < j$.

$$w(PB_{0,1}^{(g,u)}) = w(F_B^{(g,u,0)})w(F_B^{(g,u,1)}) - w(Y_R^{(g,u,0)})w(Y_R^{(g,u,1)}) = 2 \cdot 0 - \frac{1}{3} \cdot 0 = 0.$$

Similarly,

$$\text{w(PB}_{0,2}^{(g,u)}) = \tfrac{10}{3}, w(PB_{0,3}^{(g,u)}) = 0, w(PB_{1,2}^{(g,u)}) = 0, w(PB_{1,3}^{(g,u)}) = 0, w(PB_{2,3}^{(g,u)}) = 0.$$

We recall that,

$$\begin{aligned}
w(U^{(g,u)}) &= \sum_{i<j} \sum_{k<l; k,l \notin \{i,j\}} w(U^{(g,u)}_{i,j,k,l}) \\
&= \sum_{i<j} \sum_{k<l; k,l \notin \{i,j\}} w(PA^{(g,u)}_{i,j}) w(PB^{(g,u)}_{k,l}) \\
&= \sum_{i<j} w(PA^{(g,u)}_{i,j}) (\sum_{k<l; k,l \notin \{i,j\}} w(PB^{(g,u)}_{k,l})) \\
&= \sum_{i<j} w(PA^{(g,u)}_{i,j}) (SB^{(g,u)} - SB^{(g,u)}_i - SB^{(g,u)}_j + w(PB^{(g,u)}_{i,j}))
\end{aligned}$$

We determine $SB^{(g,u)}$ and $SB^{(g,u)}_i$ for all i.

$$\begin{aligned}
SB^{(g,u)} &= \sum_{k<l} w(PB^{(g,u)}_{k,l}) \\
&= w(PB^{(g,u)}_{0,1}) + w(PB^{(g,u)}_{0,2}) + w(PB^{(g,u)}_{0,3}) + w(PB^{(g,u)}_{1,2}) + w(PB^{(g,u)}_{1,3}) + w(PB^{(g,u)}_{2,3}) \\
&= 0 + \frac{10}{3} + 0 + 0 + 0 + 0 = \frac{10}{3}
\end{aligned}$$

$$\begin{aligned}
SB^{(g,u)}_0 &= \sum_{k<l; (k=0) \vee (l=0)} w(PB^{(g,u)}_{k,l}) \\
&= w(PB^{(g,u)}_{0,1}) + w(PB^{(g,u)}_{0,2}) + w(PB^{(g,u)}_{0,3}) = 0 + \frac{10}{3} + 0 = \frac{10}{3}
\end{aligned}$$

Similarly,

$$SB^{(g,u)}_1 = 0, SB^{(g,u)}_2 = \frac{10}{3}, SB^{(g,u)}_3 = 0$$

With these values, we compute $\sum_{k,l \notin \{i,j\}} w(U^{(g,u)}_{0,1,i,j})$ for each $i < j$.

$$\begin{aligned}
\sum_{k,l \notin \{0,1\}} w(U^{(g,u)}_{0,1,k,l}) &= w(PA^{(g,u)}_{0,1})(SB^{(g,u)} - SB^{(g,u)}_0 - SB^{(g,u)}_1 + w(PB^{(g,u)}_{0,1})) \\
&= 1 \cdot (\frac{10}{3} - \frac{10}{3} - 0 + 0) = 0
\end{aligned}$$

Similarly,

$$\begin{aligned}
&\sum_{k,l \notin \{0,2\}} w(U^{(g,u)}_{0,2,k,l}) = 0, \sum_{k,l \notin \{0,3\}} w(U^{(g,u)}_{0,3,k,l}) = 0, \sum_{k,l \notin \{1,2\}} w(U^{(g,u)}_{1,2,k,l}) = 0 \\
&\sum_{k,l \notin \{1,3\}} w(U^{(g,u)}_{1,3,k,l}) = \frac{5}{3}, \sum_{k,l \notin \{2,3\}} w(U^{(g,u)}_{2,3,k,l}) = 0
\end{aligned}$$

Finally, we compute $w(U^{(g,u)})$.

$$\begin{aligned} w(U^{(g,u)}) = & \sum_{k,l \notin \{0,1\}} w(U^{(g,u)}_{0,1,k,l}) + \sum_{k,l \notin \{0,2\}} w(U^{(g,u)}_{0,2,k,l}) + \sum_{k,l \notin \{0,3\}} w(U^{(g,u)}_{0,3,k,l}) + \\ & \sum_{k,l \notin \{1,2\}} w(U^{(g,u)}_{1,2,k,l}) + \sum_{k,l \notin \{1,3\}} w(U^{(g,u)}_{1,3,k,l}) + \sum_{k,l \notin \{2,3\}} w(U^{(g,u)}_{2,3,k,l}) \\ = & 0 + 0 + 0 + 0 + \frac{5}{3} + 0 = \frac{5}{3} \end{aligned}$$

As u is the only internal node with polytomy,

$$w(U^{(g)}) = w(U^{(g,u)}) = \frac{5}{3}$$

Thus, our final score for the bipartition is,

$$Score(A, B, \mathcal{G}) = 2w(S^{(g)}) - w(S^{(g)} \cup V^{(g)} \cup U^{(g)}) + w(U^{(g)}) = 2 \cdot 14 - 36 + \frac{5}{3} = -\frac{19}{3}$$

2.3 Weight normalization

Suppose a dummy taxon X has k real taxon under it. As gene trees contain only real taxa, we consider X to be involved in a quartet if any of the real taxa under it participates in that quartet. As a result, X can form k times as many quartets as a real taxon $a \in R_A \cup R_B$ with the real taxa in a set $R \subseteq \mathcal{X}$ (assuming $a \notin R$, $X_R \cap R = \emptyset$). Since our scoring is based on the sum of weights of quartets (described in Section 2.5 of the main paper), a dummy taxon gets prioritized in maximizing the score if no normalization is done (that is, all real taxa are assigned unit weight). Therefore, we attempt to ensure that a real taxon and a dummy taxon contribute equally to the score calculation.

For weight normalization, we can follow two approaches. One is to simply assign equal weights to all the real taxa under a dummy taxon. That is, if a dummy taxon d has k real taxa under it, all of them are assigned weight $\frac{1}{k}$. Another approach is to assign weights according to the "dummy taxon tree structure" described in Section 2.3 of the main paper. These weighting mechanisms (uniform and non-uniform normalizations) were proposed and used in TREE-QMC [Han and Molloy, 2023]. The latter approach (i.e., non-uniform normalization) produced superior results in all our experiments and thus further supports the observations made in the TREE-QMC study.

2.4 Gain calculation

We recall that the wQFM algorithm iteratively improves bipartition by transferring taxa from one partition to the opposite. The details of the procedure is described in [Reaz et al., 2014]. For a taxon $a \in A \cup B$, the corresponding gain, G_a, is the change in the score if a is transferred to the opposite partition. That is, if $a \in A$, $G_a = Score(A \backslash \{a\}, B \cup \{a\}, \mathcal{G}) - Score(A, B, \mathcal{G})$. Our algorithm requires us to calculate the gains of all taxa present in A and B. To do so, we calculate the change of $w(S^{(g,u)})$ at each internal node u for each taxon $a \in A \cup B$ if it is transferred to the opposite partition. We also calculate

the change of $w(U^{(g,u)})$ at each polytomy node u and the change of $w(S^{(g)} \cup V^{(g)} \cup U^{(g)})$. From there, we determine the gain for each taxon.

We can be more efficient when dealing with gains of real taxa. We utilize the fact that the change in $w(S^{(g,u)})$ is the same for transferring any $a \in R_A$ within a component $C_i^{(g,u)}$ because all such a has a unit weight. Thus, we visit each internal node u, calculate,, and store the change of $w(S^{(g,u)})$ for each of its components. Then, these changes can be accumulated using a linear graph traversal to compute G_a for all $a \in R_A$. Similarly, we can compute G_a for all $a \in R_B$.

3 Time complexity

Let n be the number of taxa present in the gene trees, r and d be the number of real taxa and dummy taxa present in a subproblem, respectively. Let (A, B) be the initial bipartition for the subproblem. Then, $r = |R_A| + |R_B|$ and $d = |D_A| + |D_B|$. We assume that the degree of the internal nodes is bounded by a constant c.

First, we can compute $w(F_A^{(g,u,i)})$, $w(F_B^{(g,u,i)})$ and $w(X_R^{(g,u,i)})$ for all u in $O(nd)$ for a tree g. Therefore, for k gene trees, the time complexity is $O(knd)$.

Then, for an internal node u of a gene tree g, we can compute $w(PA_{i,j}^{(g,u)})$, $w(PB_{i,j}^{(g,u)})$, $w(PB_k^{(g,u)})$, $\sum_{X \in D_A} w(X_R^{(g,u,i)}) w(X_R^{(g,u,j)})$ and $\sum_{X \in D_B} w(X_R^{(g,u,i)}) w(X_R^{(g,u,j)})$ for $1 \leq i, j, k \leq \deg(u)$ in $O(\deg(u)^2 d)$. Thus, for the whole set of gene trees, we can perform this computation in $O(c^2 dnk)$ time. After that, we can calculate $w(S^{(g,u)})$ in $O(deg(u))$. Then, $\sum_{g \in \mathcal{G}} w(S^{(g,u)})$ can be computed in $O(cnk)$.

Now, we can calculate $w(S^{(g)} \cup V^{(g)} \cup U^{(g)})$ from $\mathcal{X}^{(g)}$ in $O(n)$ complexity. Overall, it takes $O(nk)$ for k gene trees. We can compute $w(U^{(g,u)})$ in $O(deg(u)^2)$. Thus, $\sum_{g \in \mathcal{G}} \sum_{u \in g} w(U^{(g,u)})$ can be computed in $O(c^2 nk)$ time.

The complexity of gain computation is dominated by the gain computation of dummy taxa. We can calculate gains of all dummy taxa in $O(c^2 nkd)$. An iteration of the FM algorithm consists of transferring r real taxa and d dummy taxa. After each transfer, the gain is recalculated. Therefore, the complexity of an iteration is $O((r + d)c^2 nkd)$. The FM algorithm may take multiple iterations before finding the final bipartition where the maximum cumulative gain is non-negative. We assume that the number of iterations to find the final bipartition is bounded by a parameter α. Empirically, we have found α to be a very small number ($\alpha \leq 5$) even though we experimented exhaustively with a varying number of taxa, ranging from 37 to 2000, and other model parameters.

Therefore, given a bipartition, the total time complexity for improving the bipartition through the FM algorithm is $O(\alpha(r + d)c^2 nkd)$.

Now, for the initial bipartition, the creation of the greedy consensus tree from the gene trees at the beginning of the algorithm requires $O(n^2 k)$ time. We need to consider at most $O(n)$ edges of the consensus tree for a subproblem where we construct candidate initial bipartitions from each of the edges and calculate the score for each of them. Each score calculation step takes $O(c^2 nkd)$ time. Therefore, creating an initial bipartition takes $O(c^2 n^2 kd)$ time in total.

Thus, handling each subproblem takes $O(c^2 n^2 kd + \alpha(r + d)c^2 nkd)$ time. Since, in the worst case, $O(r + d) = O(n)$, we have the final time complexity of $O(\alpha c^2 n^2 kd)$ for a subproblem.

3.1 Time complexity for fully resolved gene trees with the assumption of balanced partitioning

For unrooted fully resolved or binary gene trees, $c = 3$. We consider the case where each bipartition is made as evenly as possible. That is, if $|A| + |B|$ is even, then $|A| = |B|$. Otherwise, $|A| - |B| = \pm 1$. Then, the height of the subproblem tree (Example in Figure 1) is $O(\log n)$. Since a dummy taxa is introduced at each level, a subproblem can have at most $O(\log n)$ dummy taxa, that is $d = O(\log n)$. Therefore, the complexity of handling a subproblem is $O(\alpha n^2 k \log n)$. We note that each divide step in our algorithm constructs an edge of the output species tree in the corresponding conquer step. Therefore, our algorithm has $\Theta(n)$ divide steps. Each divide step increases the number of subproblems by 1. Therefore, we have $\Theta(n)$ subproblems, and the total time complexity is $O(\alpha n^3 k \log n)$ or $O(n^3 k \log n)$ since empirically α is very small as mentioned before.

3.2 Time complexity without the assumptions

We have proved the time complexity of $O(n^3 k \log n)$ under the assumptions of balanced bipartitions and fully resolved gene trees. Without the assumption of balanced partitioning, we obtain a complexity of $O(n^4 k)$. Finally, the algorithm may experience a notable increase in running time for gene trees with polytomies, particularly when confronted with a large amount of polytomy. However, we do not observe a drastic rise in time in most of the cases, as shown in Table 4. Nevertheless, we can readily address the issue of polytomy by resolving them and then applying wQFM-TREE on fully resolved gene trees. This approach yields equivalent performance to wQFM-TREE without resolving polytomy (Figure 4).

4 Additional results

4.1 Comparing wQFM-TREE with wQFM

wQFM-TREE performs the same calculations as wQFM in terms of scoring candidate bipartitions and associated steps, but directly from the gene trees by circumventing the need for explicitly generating $\Theta(n^4)$ quartets. We made an effort to make wQFM scalable without sacrificing accuracy. Nevertheless, wQFM-TREE might not be the same as wQFM in terms of the initial bipartition and the normalization process. As a result, the accuracy of wQFM-TREE may differ from wQFM. However, our findings on the 37-taxon and 48-taxon datasets presented in Fig. 3 show that wQFM-TREE is generally as good as wQFM. Out of 240 data replicates across various model conditions in the 37-taxon dataset, wQFM and wQMF-TREE generated identical trees in 238 replicates, differing in only two instances. Conversely, on the 48-taxon dataset, wQFM-TREE exhibited inferior performance in two specific model conditions, which are 0.5X-1000gt-500bp and 1X-1000gt-500bp. However, no statistically significant differences were observed between them in the remaining five model conditions.

In terms of running time, quartet generation requires a significant amount of time in the case of wQFM. On the 37-taxon dataset (1X-800gt-500bp model condition), wQFM requires approximately 140 seconds to generate quartets, followed by just 4 seconds to

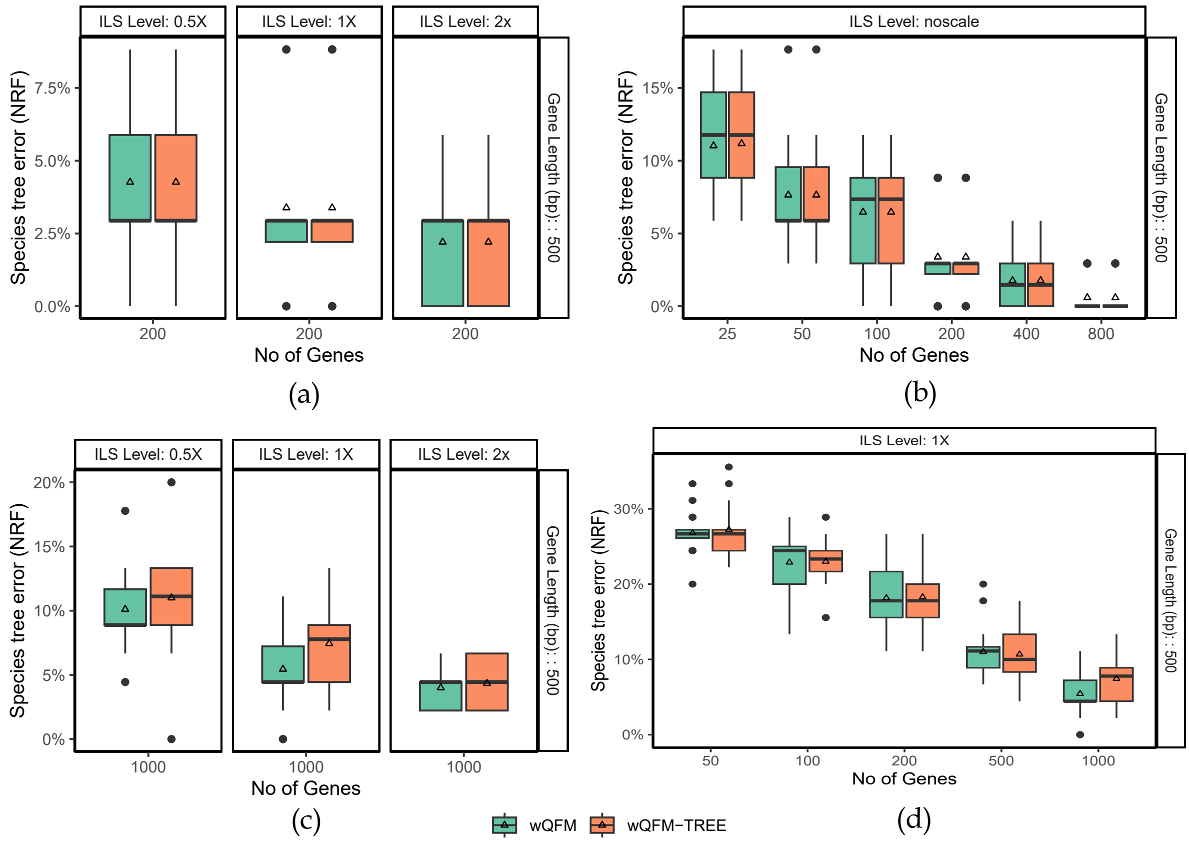

Figure 3: **Comparison of wQFM and wQFM-TREE on 37-taxon mammalian and 48-taxon avian simulated datasets.** We show the average RF rates with standard error bars over 20 replicates. (a and b) Results on the 37-taxon dataset. (a) The level of ILS was varied from 0.5X (highest) to 2X (lowest) amount, keeping the sequence length fixed at 500 bp and the number of genes at 200. (b) The number of genes was varied from 25 to 800, with 500 bp sequence length and noscale ILS. (c and d) Results on the 48-taxon dataset with varying levels of ILS and varying numbers of genes, respectively.

estimate a tree from the weighted quartets. In contrast, wQFM-TREE estimates species trees directly from the gene trees in only 4 seconds. For the 48-taxon dataset, wQFM takes about 560 seconds to generate weighted quartet distributions and an additional 14 seconds to estimate species trees from these weighted quartets. Conversely, wQFM-TREE takes 27 seconds to estimate species trees directly from the gene trees.

4.2 Handling polytomies

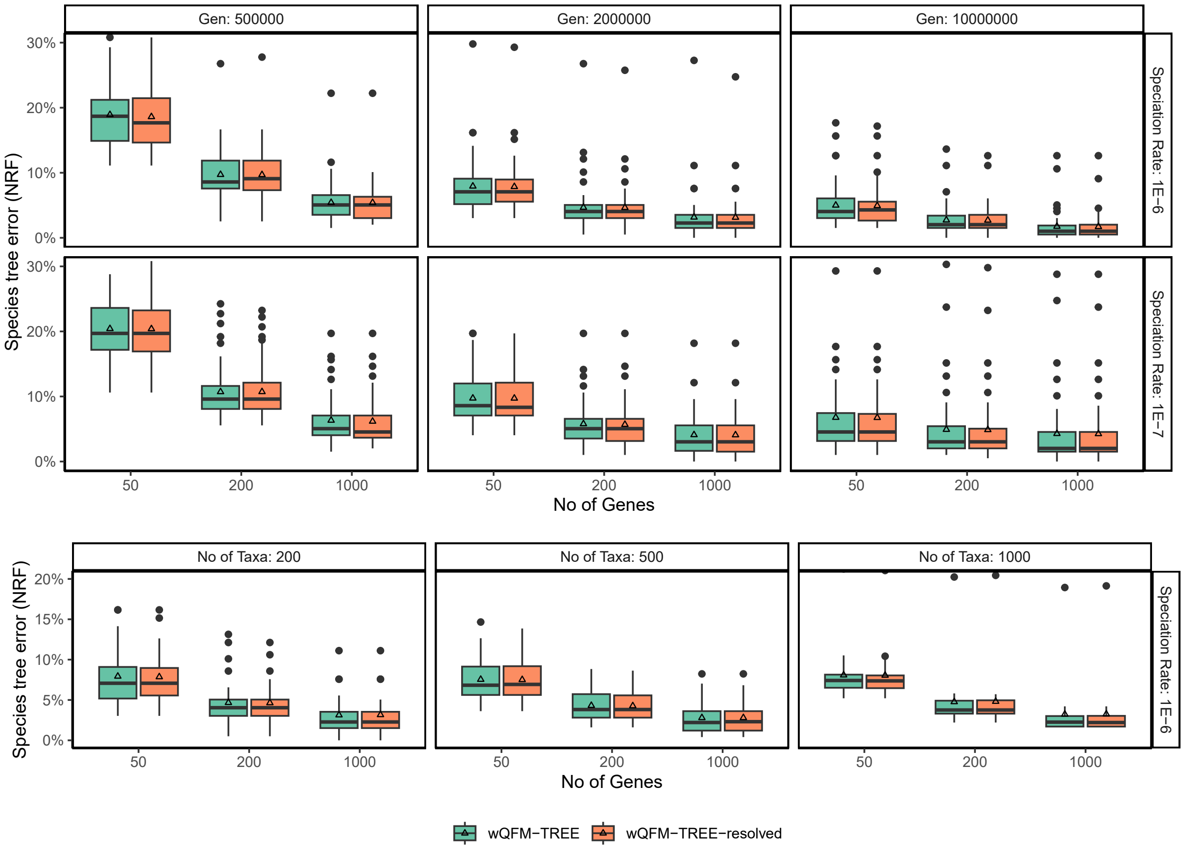

Figure 4: **Performance of wQFM-TREE with and without resolving polytomies.** (Top) Two hundred taxa and varying tree shapes and number of genes. (Bottom) Varying numbers of taxa and genes and the tree shape fixed to 2 M/1e-6.

4.3 Results of statistical tests

Table 1: Results of statistical significance tests (p-values) between wQFM-TREE and ASTRAL-III, and wQFM-TREE and TREE-QMC for the 200-taxon dataset (see Figure 2 in the main text).

No. of Generations	Speciation rate	No. of Gene Trees	p-value ASTRAL-III	p-value TREE-QMC
10000000	1E -7	50	0.0006	0.435
		200	0.002	0.280
		1000	3.68e-05	0.0034
10000000	1E -6	50	0.02	0.623
		200	0.0009	0.036
		1000	0.047	0.413
2000000	1E -7	50	0.023	0.948
		200	0.053	0.364
		1000	0.03	0.135
2000000	1E -6	50	0.0008	0.102
		200	0.0048	0.033
		1000	0.021	0.528
500000	1E -7	50	0.04	0.483
		200	0.807	0.295
		1000	0.027	0.655
500000	1E -6	50	0.023	0.56
		200	0.506	0.91
		1000	0.502	0.044

Table 2: Results of statistical significance tests (p-values) between wQFM-TREE and ASTRAL-III, and wQFM-TREE and TREE-QMC for the analysis with varying number of taxa (see Figure 3 in the main text).

No. of Taxa	Speciation Rate	No. of Gene Trees	p-value ASTRAL-III	p-value TREE-QMC
200	1E -6	50	0.0008	0.102
		200	0.0048	0.033
		1000	0.021	0.528
500	1E -6	50	4.88e-05	0.703
		200	6.3e-07	0.356
		1000	1.49e-05	0.971
1000	1E -6	50	0.0028	0.856
		200	0.007	0.609
		1000	0.0055	0.538

4.4 RF distances between clades in the wQFM-TREE and ASTRAL results for the plant dataset

Table 3: RF distances between individual clades of wQFM-TREE and ASTRAL-III with four or more taxa.

**Clade Name**	**RF Distance**
Core Rosids	0.104
Saxifragales	0
Vitales	0
Santalales	0
Caryophyllales	0
Asterids	0.0325
Proteales	0
Ranunculids	0
Magnolids	0.043
Monocots	0.009
Pinaceae	0
Cupressales	0.017
Cycads and ginkgo	0
Polypodiidae	0
Ophioglossidae	0
Lycophytes	0
Liverworts	0.15
Mosses	0.05
Hornworts	0
Zygnomophyceae	0.114
Klebsormidiales	0
Chlorophyta	0.060
Glaucophyta	0
Rhodophyta	0.111
Outgroup	0

4.5 Running time and memory consumption

Table 4: **Runtime (in seconds) of various methods on the datasets analyzed in this study.** We show the running time of wQFM-TREE with and without resolving polytomies in the gene trees. Values are shown as average (over 10 replicates for 200- and 500-taxon and 5 replicates for 1000-taxon) ± standard deviation.

No. of taxa	No. of genes	TREE-QMC	ASTRAL	wQFM-TREE resolved	wQFM-TREE unresolved
200	50	1.8±0.1	4.3±1.2	15.1±1.5	20.1±3.8
200	200	7.4±0.5	19.8±3.5	76.6±3.5	93.9±6.9
200	1000	37.1±1.2	297.6±15.8	391.1±11.1	439.5±13.4
500	50	11.4±0.6	24.4±3.6	155.3±4.6	156.6±5.9
500	200	43.8±1.0	109.1±8.9	584.5±10.1	793.5±23.4
500	1000	225.7±2.6	1509.4±34.4	2451.9±19.2	3413.7±45.1
1000	50	49.8±1.1	157.5±10.4	898.4±19.5	943.2±19.
1000	200	188.0±2.3	658.9±22.5	2786.8±34.37	3155.8±36.9
1000	1000	913.9±5.0	7336.9±71.4	11680.4±69.9	12275.2±69.7
2000	1000	3394	2161.4	24552	24552
Green plant data					
1178	410	415.3	3340.8	11318.7	19744.8

Table 5: Memory consumption (in MB) of wQFM-TREE, ASTRAL-III, and TREE-QMC on some of the datasets analyzed in this study.

No. of taxa	No. of genes	TREE-QMC	ASTRAL	wQFM-TREE
200	1000	59.195	3177.188	2418.773
200	1000	59.344	3166.379	2443.980
200	1000	59.125	3069.484	1998.371
200	1000	59.316	3223.891	2566.805
200	1000	59.035	3074.656	2269.277
200	1000	58.996	3092.105	2495.891
500	1000	142.980	3315.461	4945.230
1000	1000	295.551	6434.473	6667.750
Green plant data				
1178	410	138.020	4625.887	5758.746

4.6 Results on the avian dataset

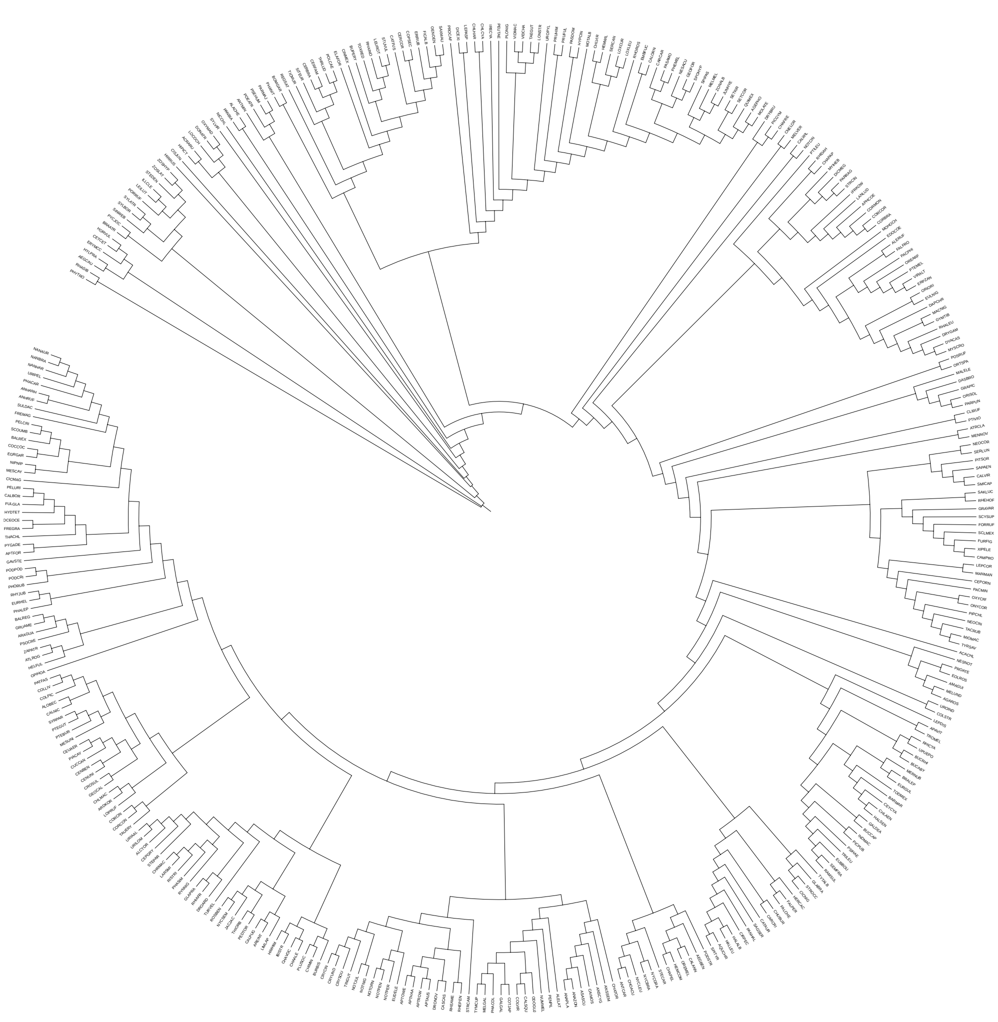

Figure 5: Phylogenetic tree reconstructed by wQFM-TREE on the avian dataset from Stiller et al. [2024].

References

C. Fiduccia and R. Mattheyses. A linear-time heuristic for improving network partitions. In *19th Design Automation Conference*, pages 175–181, June 1982. doi: 10.1109/DAC.1982.1585498.

Y. Han and E. K. Molloy. Improving quartet graph construction for scalable and accurate species tree estimation from gene trees. *Genome Research*, gr.277629.122., May 2023. ISSN 1549-5469. doi: 10.1101/gr.277629.122.

R. Reaz, M. S. Bayzid, and M. S. Rahman. Accurate phylogenetic tree reconstruction from quartets: A heuristic approach. *PLoS One*, 9(8):e104008, 2014.

J. Stiller, S. Feng, A.-A. Chowdhury, I. Rivas-González, D. A. Duchêne, Q. Fang, Y. Deng, A. Kozlov, A. Stamatakis, S. Claramunt, J. M. T. Nguyen, S. Y. W. Ho, B. C. Faircloth, J. Haag, P. Houde, J. Cracraft, M. Balaban, U. Mai, G. Chen, R. Gao, C. Zhou, Y. Xie, Z. Huang, Z. Cao, Z. Yan, H. A. Ogilvie, L. Nakhleh, B. Lindow, B. Morel, J. Fjeldså, P. A. Hosner, R. R. da Fonseca, B. Petersen, J. A. Tobias, T. Székely, J. D. Kennedy, A. H. Reeve, A. Liker, M. Stervander, A. Antunes, D. T. Tietze, M. F. Bertelsen, F. Lei, C. Rahbek, G. R. Graves, M. H. Schierup, T. Warnow, E. L. Braun, M. T. P. Gilbert, E. D. Jarvis, S. Mirarab, and G. Zhang. Complexity of avian evolution revealed by family-level genomes. *Nature*, 629(8013):851–860, May 2024. ISSN 1476-4687. doi: 10.1038/s41586-024-07323-1. URL `https://doi.org/10.1038/s41586-024-07323-1`.
